# *Allium cepa* L. Peels: Phytochemical Characterization and Bioactive Potential in Infectious and Metabolic Contexts (In Vitro, In Vivo, and In Silico)

**DOI:** 10.3390/pharmaceutics18040476

**Published:** 2026-04-13

**Authors:** Aziz Drioiche, Bshra A. Alsfouk, Omkulthom Al kamaly, Laila Bouqbis, Abdelhakim Elomri, Touriya Zair

**Affiliations:** 1 Higher Institute of Nursing and Health Techniques of Fez, Regional Health Directorate Fez-Meknes, El Ghassani Hospital, Fez 30000, Morocco; 2 Research Team of Chemistry of Bioactive Molecules and the Environment, Laboratory of Innovative Materials and Biotechnology of Natural Resources, Faculty of Sciences, Moulay Ismaïl University, B.P. 11201 Zitoune, Meknes 50070, Morocco; 3 Department of Pharmaceutical Sciences, College of Pharmacy, Princess Nourah bint Abdulrahman University, P.O. Box 84428, Riyadh 11671, Saudi Arabia; baalsfouk@pnu.edu.sa (B.A.A.); omalkmali@pnu.edu.sa (O.A.k.); 4 Ecosystems and Environmental Sciences, Faculty of Applied Sciences, Ibn Zohr University, Agadir 80000, Morocco; l.bouqbis@uiz.ac.ma; 5 INSA Rouen Normandy and CNRS, Laboratory of Organic, Bioorganic Chemistry, Reactivity and Analysis (COBRA-UMR 6014), Medical University of Rouen Normandy, 76000 Rouen, France; hakim.elomri@univ-rouen.fr

**Keywords:** *Allium cepa* L., isorhamnetin, quercetin 4′-*O*-glucoside, NADPH oxidase, anticoagulant activity, *Enterobacter cloacae*, *Pseudomonas aeruginosa*, α-amylase, α-glucosidase, OGTT, MM-PBSA

## Abstract

**Background/Objectives**: Onion (*Allium cepa*) peems are an underutilized by-product rich in polyphenols. This study evaluated the physicochemical profile, and bioactive potential (antidiabetic, antimicrobial, antioxidant, and anticoagulant) of Moroccan red onion peels using integrated in vivo, in vitro, and in silico approaches. **Methods**: Moisture, pH, ash content, and mineral elements were determined, followed by phytochemical screening and three extractions: decoction E0, aqueous Soxhlet E1, and hydroethanolic Soxhlet E2 (70/30; ethanol/water, *v*/*v*). The measurement of polyphenols, flavonoids, and tannins was carried out using colorimetric methods, while the molecular profile was studied by high-performance liquid chromatography coupled to ultraviolet detection and electrospray ionization mass spectrometry (HPLC/UV-ESI-MS). Biological activities were determined using 2,2-diphenyl-1-picrylhydrazyl, ferric reducing antioxidant power, and total antioxidant capacity assays (in vitro antioxidant); microdilution (antimicrobial); prothrombin time and activated partial thromboplastin time (anticoagulant); and α-amylase/α-glucosidase enzymatic inhibition and oral glucose tolerance tests on normoglycemic rats. Also, acute toxicity was evaluated, and molecular interactions between these proteins and ligands (docking, molecular dynamics, and MM-PBSA) were analyzed. **Results**: Physicochemical analyses showed an acidic pH (3.06) and high ash content (15.21%), with the concentration of regulated elements remaining within FAO/WHO limits. The extractive content was between 6.90% E0 and 19.18% E2. The E1 extract had the maximum amount of total polyphenols (178.95 mg GAE/g); on the other hand, E2 was the richest in flavonoids by 121.43 mg QE/g. The HPLC/ESI-MS analysis of E0 revealed 20 compounds, among which flavonoids (84.93%) were predominant, with isorhamnetin (30.26%), followed by quercetin and its glycosylated forms. E1 showed the most potent antioxidant effects (IC_50_ DPPH, 22.38 µg/mL, as that of ascorbic acid). The antibacterial activity of E0 was especially potent towards *Enterobacter cloacae* and *Pseudomonas aeruginosa* (MIC 75 µg/mL). A mild dose-dependent anticoagulant effect was seen. Antidiabetic activity was found to be outstanding: α-amylase (IC_50_ 62.75 µg/mL) and α-glucosidase (IC_50_ 8.49 µg/mL, stronger than acarbose) inhibitions were corroborated in vivo by a considerable decrease in the glycemic area under the curve. The molecular docking study in silico demonstrated strong molecular interactions, especially for quercetin 4′-*O*-glucoside with good binding energies. **Conclusions**: *A. cepa* peels from Morocco can be considered a safe plant matrix containing bioactive flavonoids with strong antioxidant and selective antimicrobial activities and promising antidiabetic effects, supported by molecular modeling.

## 1. Introduction

*Allium* is one of the largest plant genera, with around 1100 recognized species, including *Allium cepa* L. (onion) [1]. This plant belongs to the Amaryllidaceae family, and its name is derived from the Latin word “unio,” which means “single” or “one”; this is due to its production of a singular bulb [2]. Its most common name aside from just “onion” is bulb onion or common onion, while the latter is also applied to related plants. This is because it has been cultivated for centuries and traveled through continents [3].

The onion (*Allium cepa* L.) has one of the highest horticultural crop yields in the world. It is grown all over the world for both fresh and processed products (for example, dehydrated and other value-added uses). Worldwide, onions are grown over a total of approximately 4.5 million hectares, producing some 92.1 million tons of onions with an average global yield of 19.3 t·ha^−1^ [4]. India is currently the biggest onion producer, with Egypt, the USA, China, and Turkey also being amongst the top. Productivity-wise, the Republic of Korea, with the highest average yield (79.6 t·ha^−1^), is followed by the USA (71.1 t·ha^−1^) [5].

The chemical profile of the species comprises predominantly flavonoids (mainly quercetin and kaempferol), organosulfur compounds (thiosulfinates and allyl sulfides), anthocyanins, phenolic acids, steroidal saponins, and inulin-type fructans [6,7,8]. Quercetin, reported as a major constituent in peels, is known for its radical scavenging capacity and its ability to modulate inflammatory pathways [9]. Organosulfur compounds, however, have potent inhibitory activity against many microorganisms, which include not only a large number of bacteria but also fungi [10,11]. Extracts of *A. cepa* peels, which are generally regarded as agro-industrial waste, exhibit various biologically relevant activities such as antioxidant activity and antimicrobial effect against important strains like methicillin-resistant *Staphylococcus aureus* (MRSA), *Candida albicans*, *Pseudomonas aeruginosa*, and *Escherichia coli*, along with the capability to modulate glycemic homeostasis/lipid profiles [12,13,14].

The culture of *A. cepa* in the Maghreb region is indeed very ancient and represents key crops that fit into local food security strategies. Morocco currently has over 25,000 hectares of this crop, which is planted in diverse agroecological zones ranging from the Atlantic plains to pre-Saharan oases. It generates approximately 850,000 tons of food per year, and the peels represent an estimated 10 to 15% of the total biomass of the bulb [15]. These peels are commonly discarded, yet their valorization fits circular-economy strategies by converting an abundant agro-industrial residue into a high-value source of health-related phytochemicals [15]. This is particularly relevant in Morocco, located in a Mediterranean biodiversity hotspot that may harbor distinctive genetic resources of *A. cepa* [16,17].

Despite the broad international evidence supporting the bioactivities of *A. cepa* peels, Moroccan-grown red onion peels remain insufficiently characterized using standardized and integrative approaches that relate composition to function. In particular, studies are still lacking that jointly establish physicochemical quality parameters, systematically map the phytochemical profile, and connect this composition to major bioactivities through an integrated in vitro, in vivo, and in silico validation workflow.

We hypothesized that extraction conditions shape the phenolic/flavonoid fingerprint of red Moroccan onion peels and thereby determine the intensity and selectivity of their biological activities. Accordingly, we expect decocted, aqueous, and hydroethanolic extracts to produce distinct metabolite profiles associated with measurable antioxidant, selective antimicrobial, antidiabetic, and anticoagulant effects, with molecular modeling supporting plausible interactions between major peel flavonoids and relevant molecular targets.

This study was designed to provide an end-to-end assessment of Moroccan red onion peels by establishing physicochemical quality indices and mineral composition; comparing three extraction strategies and quantifying total phenolics, flavonoids, and tannins; profiling major constituents using HPLC-UV coupled with ESI-MS; and linking the resulting extracts to functional outcomes through complementary antioxidant, antimicrobial, antidiabetic, and anticoagulant evaluations, integrating in vitro assays with in vivo testing. Acute toxicity was assessed to document short-term safety, and in silico interaction analyses were performed to support mechanistic interpretation of the experimental findings.

## 2. Materials and Methods

### 2.1. Vegetal Material

The *A. cepa* peels utilized in this study were gathered from onion fields located in the Boulemane region (Guigou municipality, Fez-Meknes, Morocco) during April 2025. Table 1 provides extensive details about the source, parts of the plants collected, and their harvesting location. The cultivar/variety name was not provided by the farmer/supplier and could not be verified; therefore, the collected material consisted of the dry outer red onion peels (papery external tunics). Peels were collected in clean paper bags and air-dried at ambient temperatures in the shade of a well-ventilated area for 10 days after collection. Finally, the dried material was powdered with a standard electric grinder to obtain a fine homogeneous powder and kept in airtight light- and humidity-proofed amber containers at room temperature until further analyses. The species was formally identified botanically at Morocco’s Laboratory of Botany and Plant Ecology, part of the Scientific Institute Rabat.

### 2.2. Extraction of Phenolic Compounds

Decoction and solid–liquid extraction utilizing a Soxhlet apparatus were employed to enhance the recovery of bioactive compounds, including phenolic chemicals. Thirty grams of plant material was decocted for one hour at 80 °C in 600 mL of distilled water to extract phenolic compounds. Following a five-minute incubation of the mixture, the insoluble residue was filtered using reduced pressure. The resultant extract was preserved in a glass vial for future application following the drying of the filter in an oven maintained at 70 °C. Thirty-gram samples were extracted using a Soxhlet apparatus with 300 mL of distilled water and a hydroethanolic solution (70:30, *v*/*v*). Prior to utilizing the extracts, they were concentrated using a rotary evaporator following multiple extraction cycles to remove surplus solvent and enhance chemical purity. Table 2 provides a comprehensive encoding of the manually extracted excerpts from this trial.

### 2.3. Selection of Animals for Research Purposes

Outbred Swiss albino mice (*Mus musculus*) of both sexes (7 weeks old; 20–35 g) were employed in the acute toxicological study, while Wistar rats (*Rattus norvegicus*) of both sexes (10 weeks old; typically 190–240 g in the males and slightly less than that in females) were employed to evaluate the in vivo antidiabetic efficacy. All animals were sourced from the animal room of the biology department, Faculty of Sciences Dhar El Mehraz, Sidi Mohamed Ben Abdallah University (Fez, Morocco), where they were maintained under standardized laboratory conditions (22 ± 2 °C; 12 h light/12 h dark cycle), with ad libitum access to standard rodent chow and drinking water. All experimental procedures were approved by the local ethics committee (04/2019/LBEAS) and followed all animal welfare-related principles, as well as applicable ethical and legal regulations [18].

### 2.4. Quality Control of Plant Specimens

#### 2.4.1. Moisture Content

The AFNOR standard (NF-V03-402, 1985) indicated that the moisture was noncompliant [19]. A precisely measured 5 g subsample of the plant material was placed in pre-dried and tared crucibles. After a 24 h drying period at 103–105 °C, the crucibles containing the plant material were placed in a furnace. Subsequent to cooling in a desiccator, the crucibles were reweighed. The subsequent Equation (1) was employed to ascertain the moisture content:

(1) $$MC\%=\left(\frac{m_{0}-m_{1}}{m_{0}}\right)\times 100$$

where *m*_0_ represents the starting mass of the plant in grams (g), and *m*_1_ denotes the mass after drying in grams (g). The product is determined as a percentage based on the weight of dry materials.

#### 2.4.2. Assessment of pH

The pH value of the product is used to determine how acidic it is. The procedure involves combining 10 milliliters of warm distilled water with 2.0 g of the sample. After filtration, the mixture is permitted to cool before the electrode is immersed in an adequate proportion of the filtrate to assess the pH [20].

#### 2.4.3. Ash Content

Ash content was measured according to NF ISO 5984 [21]. In brief, 5.0 g of the powdered plant was transferred to an empty evaporating dish and then ashed in a muffle furnace at 550 °C until the weight was constant. The crucible was weighed again after cooling in a desiccator, and ash content was calculated using Equation (2):

(2) $$OM\%=\left(\frac{m_{1}-m_{2}}{\mathrm{TE}}\right)\times 100$$

*OM*%: Organic Matter Percentage;
*m*_1_: Mass of the capsule and sample prior to calcination;
*m*_2_: Mass of the capsule and sample post-calcination;
TE: Test portion.

#### 2.4.4. Trace Element (Metals and Metalloids) Analysis by ICP–AES

Several regulated trace elements (metals and metalloids) were examined, including lead (Pb), iron (Fe), arsenic (As), antimony (Sb), cadmium (Cd), titanium (Ti), and chromium (Cr). Overall, the measured concentrations were within the recommended limits for plant-based materials, suggesting no relevant contamination under the investigated sourcing condition. Medications that contain active components known to naturally accumulate elevated amounts of cadmium are an exception. The AFNOR standard method (LST EN 15510, 2017) [22], which involved mineralization with aqua regia (HNO_3_ + 3 HCl), was followed in determining the amounts of these elements. In this manner, samples are prepared using appropriately large specimens, perhaps minimizing any loss of representativeness. The procedure involved combining 3 mL of aqua regia, which contained 1 mL of concentrated HNO_3_ (99%) and 2 mL of HCl (37%), with 0.1 g of the plant’s ideally milled dry material. After two hours of refluxing at 200 °C, the resultant mixture was left to cool and settle. The supernatant was filtered using a 0.45 µm filter, resulting in a final volume of 15 mL in distilled water. At the UATRS (Technical Support Unit for Scientific Research) CNRST Rabat laboratory, ICP-AES analysis (Ultima 2 Jobin Yvon, Longjumeau, France) was employed to quantify heavy metals [23].

### 2.5. Phytochemical Screening

This qualitative investigation aimed to identify chemical families by the analysis of dissolution, precipitation, and turbidity tests. Supplementary procedures involved examining materials under ultraviolet light and documenting certain color differences. The peels of the *A. cepa* that were the subject of the investigation underwent phytochemical examination. The methods outlined in Dohou et al. [24], Judith [25], Mezzoug et al. [26], Bekro et al. [27], Bruneton [28], and N’Guessan et al. [29] were used to make the powder of the dried plant material and to determine the various chemical groups.

### 2.6. Investigation of Phenolic Compounds

#### 2.6.1. Determination of Total Polyphenols

The total concentration of polyphenolic chemicals in the analyzed extracts was quantified using the Folin–Ciocalteu method, as outlined by Singleton and Rossi [30]. This color reaction is based on the oxidation of phenolic compounds to a distinctive blue coloration. This reduction product is based on the capacity of the Folin–Ciocalteu reagent (a mixture of phosphotungstic acid (H_3_PW_12_O_40_) and phosphomolybdic acid (H_3_PMo_12_O_40_)) to be reduced to form blue tungsten and molybdenum oxides. The absorbance values were measured at 760 nm, employing a UV-Visible spectrophotometer (model UV mini-1240, Shimadzu, Beijing China). This blank was prepared without an extract and presented as a reagent blank, which was used as a reference solution to ensure the analysis’s precision. The reference compound used was gallic acid (50 µg/mL), and a calibration curve under the same experimental conditions was established. The contents of total polyphenol were determined from a linear equation (Y = ax + b) and presented as milligrams of gallic acid equivalent per gram of dry extract (mg GAE/g). All measurements were made in triplicate to ensure reproducibility.

#### 2.6.2. Determination of Flavonoids

Flavonoid levels in the plant extracts were determined by an aluminum chloride (AlCl_3_) colorimetric assay, as described by Hung [31] and Djeridane [32]. The method is based on the formation of a stable flavonoid–AlCl_3_ complex between aluminum chloride and flavonoids possessing hydroxyl (OH) groups in their structure, which gives characteristic absorbance signals. UV spectrophotometry was used for measurements at 433 nm. Quercetin, the most popular compound of flavonoids, was used as the standard reference and assayed under the same experimental conditions as plant extracts. A calibration curve was plotted with quercetin solutions of 5–30 µg/mL based on the linear regression equation (Y = ax + b). Flavonoid contents (mg of quercetin equivalents/g extract weight (mg QE/g)) were calculated based on the dry extract. All experiments were performed in triplicate to achieve accuracy and reproducibility.

#### 2.6.3. Determination of Condensed Tannins

The concentrations of condensed tannins in the plant extracts studied were determined by measuring absorption at 500 nm using pure condensed tannin reference compounds, as described earlier, because they reflect the commonly recommended colorimetric measurement from the vanillin assay for their quantification [33]. In brief, different volumes of a (+)-catechin stock solution (2 mg/mL) were mixed with 3 mL of vanillin reagent, which was prepared in methanol to reach a final concentration of 4% (*m*/*v*). The emulsions were vigorously mixed by shaking. To each reaction and standard, 1.5 mL of concentrated hydrochloric acid was then added. The solutions were then incubated for 20 min at room temperature for chromophore generation. The absorbance at 499 nm was measured with a UV-Vis spectrophotometer, and the reagent blank was used as a reference. The tannin content of plant extracts was calculated by substituting standard catechin with sample extracts and following the above-described method. Using the standard curve, the results were expressed as milligrams of catechin equivalent per g of dry weight material (mg CE/g).

#### 2.6.4. Determination of Hydrolyzable Tannins

Hydrolyzable tannin concentration was determined according to Willis and Allen [34], with minor modifications to improve accuracy. In the assay, 10 µL of plant extracts was mixed with 5 mL of potassium iodate (KIO_3_) at 2.5% solution and vortexed for 10 s. Optimal reaction times were different for samples and standards; that is, when the absorbance maximum of plant extracts (2 min) was reached, those from an equimolar tannic acid reference solution needed 4 min, suggesting distinct kinetic characteristics between the two matrices. After incubation, absorbance was read at 550 nm using a UV-VIS spectrophotometer. Standard solutions of tannic acid in the range of 100–2000 µg/mL (eleven) were selected to generate a calibration curve. The hydrolyzable tannin concentration was determined by the standard curve established with these concentrations and expressed as milligrams of tannic acid equivalent per gram of dry plant material (mg TAE/g).

#### 2.6.5. HPLC/UV ESI-MS Analysis of *A. cepa* Extracts

High-performance liquid chromatography (HPLC) was used to identify the phenolic profile of *A. cepa* decoction in combination with Q Exactive Plus Mass Spectrometry and Electrospray Ionization (Q Exactive Plus/ESI-MS, Thermo Fisher Scientific, Sunnyvale, CA, USA), which provides high sensitivity and accuracy to define bioactive compounds. Chromatographic analysis was performed on an UltiMate 3000 UHPLC system (Thermo Fisher Scientific, Waltham, MA, USA) using a reverse-phase C18 column (250 × 4 mm, 5 μm particle size; Lichro CART, Lichrospher, Merck, Darmstadt, Germany) and kept at 40 °C. Samples were stored at 5 °C in the autosampler until they were injected.

The separation of phenolic compounds was performed on a Waters ACQUITY UPLC HSS T3 C18 column (Waters Corporation, Miford, MA, USA), with the binary mobile phases of solvent A (0.1% formic acid in water) and solvent B (0.1% formic acid in acetonitrile). The column was developed with solvent B from 2 to 95% over a 30 min run. The injection volume was 20 μL, and the flow rate of the mobile phase was 1 mL/min.

Mass spectrometric detection was conducted using a Maxis Impact HD mass spectrometer (Bruker Daltonik, Bremen, Germany) in negative electrospray ionization mode with broadband collision-induced dissociation (bbCID) for MS/MS analysis. The desolvation and nebulization gas was nitrogen. The conditions of instrument parameters were as follows: capillary voltage of 3000 V, drying gas temperature of 200 °C, drying gas flow rate of 8 L/min, nebulizer pressure of 2 bar, and plate offset of −500 V.

UV spectra were recorded using a diode array detector (Merck-Hitachi, Darmstadt, Germany) from 190 to 600 nm. Further detection of several phenolic class compounds was possible at 280, 320, and 360 nm. Mass spectra were collected in the range of *m*/*z* 100–1500, and compounds were identified using Chromeleon 7.2 software (Thermo Scientific, Waltham, MA, USA).

### 2.7. Antioxidant Activities

#### 2.7.1. Antiradical Activity by the DPPH• Assay

The free radical scavenging ability was determined by the DPPH• (2,2-diphenyl-1-picrylhydrazyl) assay [35], which assesses the electron-donating ability of antioxidant substances, notably phenolics, to convert stable DPPH• radical into a non-radical form. This reductive reaction causes a visible colour change from deep violet to light yellow, making it possible to quantify the sample spectrophotometrically. The DPPH• stock solution was prepared by dissolving 2.4 mg of DPPH• in 100 mL of ethanol, obtaining a concentration of 6 × 10^−5^ M. The plant extracts and the reference standards were also dissolved in ethanol to guarantee solvent compatibility. Then, 200 µL of sample (extracts or standard) at different concentrations was mixed with 2.8 mL of freshly prepared DPPH• solution in a test tube for each assay. The solutions were allowed to sit in the dark for 30 min at room temperature so that no photodegradation of the radicals could occur. Absorbance was measured at 515 nm by a UV-Vis spectrophotometer after incubating the solution, with pure ethanol used as a reference blank. A blank was made with DPPH• solution but without extracts in order to determine background absorbance. The anti-radical power was expressed as percentage inhibition by Equation (3) [36], which permitted immediate comparability of scavenging capacity among plant extracts and the reference antioxidant:

(3) $$\%\;\mathrm{AA}=\frac{\mathrm{Abs}\;\mathrm{control}\;-\;\mathrm{Abs}\;\mathrm{sample}}{\mathrm{Abs}\;\mathrm{control}}\;\times \;100$$

where the following definitions are provided: % AA: percentage of antiradical activity; Abs control: absorbance of the blank (DPPH• in ethanol); Abs sample: absorbance of the test solution (extracts).

#### 2.7.2. Ferric Reducing Antioxidant Power Method (FRAP)

The FRAP assay was used for the determination of plant extracts’ reducing power, as previously described by Oyaizu (1986) [37,38]. In this method, the reducing power is determined by the reduction of Fe^3+^ to Fe^2+^ in the presence of K_3_Fe(CN)_6_. The experimental conditions were the same as with our previous samples to allow for comparison across studies. The reducing power was measured spectrophotometrically at 700 nm using a UV-Vis spectrophotometer. The instrument was calibrated with deionized water and, as a reference baseline, a reagent blank prepared in exactly the same manner as the test samples. Ascorbic acid, a known antioxidant, was also evaluated together with the plant extracts and served as a positive control for comparison. High absorbance values are directly proportional to the higher reducing power and therefore a sign of a high antioxidant power in the samples analyzed.

#### 2.7.3. Total Antioxidant Capacity (TAC)

The total antioxidant capacity of plant extracts was assayed according to the method of Khiya [39] using a phosphomolybdenum assay. This method is based on the reduction of molybdate ions (MoO_4_^2−^, Mo(VI)) to molybdenum (V) (MoO_2_^+^) as a result of the presence of antioxidant compounds. The product is an acid-resistant, green phosphate/Mo(V) complex, the intensity of which correlates with antioxidant capacity. The reagent mix consisted of 4 mM ammonium molybdate, 28 mM sodium phosphate, and 0.6 M sulfuric acid. To this reagent solution, 0.3 mL of plant extract was poured. Reaction tubes were sealed and incubated at 95 °C for 90 min to ensure full reduction of the molybdenum complex. After cooling to ambient temperature, absorbance was recorded using a UV-Vis spectrophotometer. A reagent blank, prepared identically but without extract, served as the reference for baseline correction. Quantification was achieved through a standard calibration curve constructed using various concentrations of ascorbic acid. The results were reported as mg AAE/g CE, and this allowed for a standardized comparison of antioxidant activity between the extracts.

### 2.8. Antimicrobial Activities

#### 2.8.1. Microbial Materials

The aqueous extract of *A. cepa* was evaluated for antibacterial activity against eight fungal strains and twenty-nine bacterial strains (Table 3). These virulent pathogens are well-known for being hazardous to humans and for being highly resistant and invasive. Their participation in various infections in Morocco is a considerable clinical and therapeutic obstacle. The isolated strains were acquired from Mohamed V Meknes Hospital. Each microbial strain included in this study was revived from Sabouraud broths and Mueller–Hinton, subcultured, and preserved at −80 °C in a 20% glycerol stock solution prior to assessment of viability and repeatability of results.

#### 2.8.2. Establishing the Minimum Bactericidal, Minimum Fungicidal, and Minimum Inhibitory Strengths

Antimicrobial susceptibility was determined by the broth microdilution method, as described elsewhere [40], in 96-well culture plates. MIC is defined as the lowest test concentration of extract at which no color change occurred after incubation. The plant extract stock solutions were made in 10% DMSO, and then, the same solvent was used for dilutions. The final extract concentrations obtained ranged from 5 to 9.3 × 10^−2^ mg/mL by serial two-fold dilutions. Mueller–Hinton or Sabouraud broth was used for bacterial and fungal assays, respectively, in a final volume of 100 µL in each well. Standardized microbial inocula (100 µL) were added to provide final concentrations of 10^6^ CFU/mL for bacteria and 10^4^ CFU/mL for fungi. The resazurin colorimetric method was employed for viability enumeration of the microbes. In 24 h of incubation at 37 °C, 10 µL of resazurin solution was included in each well. A color change from purple to pink was observed after further incubation for 2 h at 37 °C, confirming microbial growth. The MIC was considered the lowest concentration displaying no color change, which suggests complete growth inhibition. Positive and negative growth controls were also added to wells 11 and 12. Each assay was carried out in duplicate. The antifungal agent terbinafine was used as a standard. A stock solution was created by dissolving 250 mg of terbinafine powder in 2 mL of 10% DMSO. To establish the bactericidal and fungicidal endpoint, 10 µL of material from those wells with no visible growth was subcultured onto Mueller–Hinton agar (bacteria) or Sabouraud agar (fungi) and incubated at 37 °C for 24 h. The MBC, or MFC, was determined as the concentration at which ≥99.99% of CFU/mL were killed compared to the starting inoculum. The type of antimicrobial activity was determined by MBC/MIC or MFC/MIC ratios [41]. Extracts were considered to be bactericidal or fungicidal when the ratio was ≤4, while they were bacteriostatic or fungistatic otherwise. All bioassays were performed following protocols recommended by the Clinical and Laboratory Standards Institute (CLSI) for antimicrobial susceptibility testing.

### 2.9. Anticoagulant Activity

The anticoagulant activity of *A. cepa* decoction was determined by two coagulation tests, PT (prothrombin time) and aPTT (activated partial thromboplastin time), with some modifications according to the method of Hmidani et al. [42]. The tested concentrations were between 0.179 and 11.5 mg/mL for dose-dependent effects of coagulation parameters. Whole blood was drawn in tubes with 3.8% trisodium citrate as an anticoagulant. Platelet-poor plasma (PPP) was evacuated after centrifugation for 10 min at 25,000 rpm. The plasma was pooled and stored at −10 °C prior to analysis. Fifty microliters of pooled citrated plasma and plant extract solution aliquots was mixed with each other and pre-incubated at 37 °C for 10 min. Then, 100 μL of PTT reagent (CKPREST^®^, Diagnostica Stago, Parsippany, NJ, USA) was added, and then, the mixture was again incubated at 37 °C for 5 min, following which coagulation was triggered by the addition of 100 µL of 25 mmol/L CaCl_2_, and clotting times were assessed instantly. PT determination involved the mixing of 50 µL of pooled citrated plasma with 50 µL of plant extract solution, and incubation was carried out at 37 °C for 10 min. Then, 200 µL of prewarmed Neoplastin^®^ Cl reagent (preincubated for 10 min at 37 °C) was added for coagulation, and the time of clotting was recorded. The clotting times of the extracts at all concentrations tested were presented in seconds. The measurements were performed six times each by an automated coagulometer (MC4Plus, MER-LIN Medical^®^, Lemgo, Germany) to achieve accuracy and reproducibility.

### 2.10. Antidiabetic Activity

#### 2.10.1. Investigation of Aqueous Extracts’ Inhibitory Effect on Pancreatic α-Amylase Activity In Vitro

The aqueous extracts were tested for α-amylase inhibitory activity according to the method described by Daoudi et al. [43], and acarbose was used as the standard inhibitor. Acarbose (1, 0.8, 0.6, 0.4, and 0.2 mg/mL) and aqueous plant extract (0.89, 0.45, 0.22, 0.11, and 0.06 mg/mL) were serially diluted with a phosphate buffer solution. A test solution (extract or acarbose) of 200 µL was mixed with a phosphate buffer in each assay. Each blank sample consisted of a 200 µL phosphate buffer without enzymes and was used to zero the absorbance. Tubes were pre-equilibrated at 37 °C and kept in liquid form for 10 min before the assay was started. After that, 200 µL of α-amylase enzyme solution and a starch substrate were added into the tube. The enzyme–substrate mixture was allowed to completely interact at 37 °C for 15 min. The reaction was stopped by the addition of 600 µL of the DNSA reagent. The colorimetric reaction was stopped by boiling for 8 min and cooling instantly on ice. After dye elution from each tube, all were diluted to 1 mL of distilled water to normalize the sample’s volume. Absorbance was recorded at 540 nm on a UV-Vis spectrophotometer with buffer-only controls used to provide a baseline for reference correction. The percentage of α-amylase inhibition was determined as shown in Equation (4):

(4) $$\%\;\mathrm{Inhibition}\;=\frac{A\;\mathrm{control}\;-\;A\;\mathrm{sample}}{A\;\mathrm{control}}\;\times \;100$$

where the following is defined: Acontrol = absorbance of enzyme reaction without inhibitor; Asample = absorbance of enzyme reaction in presence of extract or acarbose.

Inhibition of α-amylase activity indicates potential to reduce carbohydrate hydrolysis, representing a promising mechanism for glycemic control in diabetes management.

#### 2.10.2. Investigation of Aqueous Extracts’ Inhibitory Effect on α-Glucosidase Activity In Vitro

The α-glucosidase inhibitory activity of *A. cepa* aqueous extract was determined by p-nitrophenyl-α-D-glucopyranoside (pNPG) as the chromogenic substrate according to Chatsumpun et al. [44] with slight modifications. The dose–response curve was completed using a series of extract concentrations ranging from 0.488 to 100 µg/mL. All test samples were formulated in 5% DMSO as a solvent vehicle. A phosphate buffer (pH 6.8) was used for the dissolution of an α-glucosidase enzyme to keep an optimal catalytic environment. Acarbose, a well-known antidiabetic agent as an α-glucosidase inhibitor, was used as a positive control for comparison. The assay was carried out in 96-well microtiter plates. First, 40 µL of α-Glucosidase solution (0.1 U/mL) was added into each well, and then, 10 µL of the extract or control solution was added. The plate was swirled gently and pre-incubated for 10 min at 37 °C to allow interaction of the enzyme with the inhibitor. Then, 50 µL of pNPG substrate (1 mM) was then added to start the enzymatic reaction. Further incubation at 37 °C for 20 min permitted substrate hydrolysis. The reaction was stopped by the addition of 100 µL of Na_2_CO_3_ solution (0.1 M), which raises the pH value and denatures the enzyme. The reaction product, p-nitrophenol, a yellow compound produced by hydrolysis, was determined, and the absorbance was read at 405 nm in a microplate reader. The percent inhibition of α-glucosidase activity was determined using Equation (4), and the extract efficiencies toward α-glucosidase were indirectly compared with acarbose. These results can serve as preliminary scientific evidence of the use of *A. cepa* extracts in the management of postprandial hyperglycemia in diabetes mellitus.

#### 2.10.3. Study of Acute Toxicity

The present study was performed to determine the short-term safety of oral *A. cepa* aqueous extracts using an acute toxicity model in a healthy mouse as a proxy for potential human exposure. The test was performed following the Organization for Economic Co-operation and Development (OECD) Guideline 423 (Acute Oral Toxicity—Acute Toxic Class Method) [45]. Adult outbred Swiss albino mice (20–35 g) were food-deprived for 14 h before administration of the doses but had free access to water, and then, they were divided randomly into four groups (*n* = 6 per group; ♂/♀ = 1).

The negative control group received distilled water (10 mL/kg), while the treatment groups received, by oral gavage, a single oral administration of E0 at doses of 0.5, 1, and 2 g/kg (selected in accordance with OECD 423 dose levels/limit dose approach). The animals were closely observed during the first hours following dosing (up to 10 h) for any signs of toxicity (behavioral changes, physical distress, or adverse reactions), and then, they were monitored daily for 14 days for clinical and behavioral abnormalities and mortality. This period of systematic observation enabled a thorough evaluation of the extract’s safety at the tested dose levels.

#### 2.10.4. Investigation of the *A. cepa* Aqueous Extract’s Antihyperglycemic Effect in Normal Rats In Vivo

The oral glucose tolerance test (OGTT), oral sucrose tolerance test (OSTT), and assessment of postprandial blood glucose control, an important intervention for diabetes management, were used to investigate the hypoglycemic activity of *A. cepa* aqueous extract E0 in normoglycemic rats. Adult rats (200–250 g) were fasted for 14 h, and they were then randomly divided into three groups: aqueous extract (2 mL/kg; n = 6, male/female ratio = 1:1), distilled water (10 mL/kg) as a negative control, or glibenclamide (2 mg/kg) as a positive control. Animals were anesthetized with diethyl ether, and the baseline fasting blood glucose level (t_0_) was determined before oral treatment with the test substances; 30 min after treatment, blood sugar measurements were taken. An oral glucose challenge (2 g/kg) was given to mimic postprandial conditions; blood glucose values were measured at 30, 60, 90, and 150 min after glucose load. This experimental setup allowed a comparison of glycemic responses associated with the treatment groups, providing permissive evidence regarding the extract’s potential to suppress glucose absorption and manage carbohydrate metabolism in putative antidiabetic treatments.

### 2.11. Molecular Docking

Three-dimensional crystal structures of the target proteins for the listed proteins in Table 4 were downloaded from the RCSB Protein Data Bank (https://www.rcsb.org/, accessed on 16 December 2025) and visualized with UCSF Chimera (version 1.5.6; The Scripps Research Institute). The targets shown in Table 4 were chosen to mechanistically relate each of the principle onion-peel compounds that have been identified/inferred in this work (7 ligands) to the biological activities tested (antimicrobial, antioxidant, antidiabetic, and anticoagulant), prioritizing protein targets that are experimentally validated and commonly exploited players within these pathways, as well as those playing critical roles either in microbial survival and/or virulence or host-relevant mechanisms (DNA replication and cell-wall/folate/fatty-acid pathways and resistance proteins for antibacterial activity; CYP51/lanosterol demethylase for antifungal activity; redox-based oxidative-stress-related enzymes for antioxidant effects; α-amylase/α-glucosidase for postprandial glycemia; thrombin-related coagulation), with crystallographic structures available containing defined binding pockets (a preference being made for having co-crystallized a native ligand/inhibitor) amenable to structure-based docking. Accordingly, the mechanistic hypothesis we tested was that onion-peel phenolics/flavonoids could bind within the functional/catalytic pockets of these targets and thus plausibly underlie the observed bioactivities; thus, docking outputs (binding energies and interaction patterns with key pocket residues) were used as supportive, not definitive, evidence to link chemical composition with measured biological effects.

The protein preparation was carried out using Chimera and AutoDock Tools (version 1.5.6; The Scripps Research Institute), including the removal of crystallographic solvent molecules, heteroatoms, and non-binding protein chains, as well as native co-crystallized ligands from active sites to prepare an appropriate docking environment for docking simulations. Polar hydrogen atoms were then added, and Gasteiger partial charges were assigned to properly represent electrostatic interactions; all prepared protein structures were saved in PDBQT format for further analysis. Crystallographic structures of the ligands for the 7 compounds of interest were downloaded from PubChem in SDF format, and their conformational stability was attained by energy minimization and converted to PDBQT supported by OpenBabel version 3.0.1. Molecular docking simulations were conducted with AutoDock Vina using a scoring function, taking into account the binding affinity computed; for docking, a 3D grid box was generated centered in the active site of interest, and dimensions and coordinates were calculated to include all related resolved binding pocket residues. Binding affinities and ligand–receptor interactions were analyzed using PyMOL version 2.5.5, by visualizing the docking results. Protocol validation was done by removal of the co-crystallized native ligand and re-docking it into the original binding site, using the root-mean-square deviation (RMSD) value between the docked pose and crystallographic conformation to evaluate the docking method’s accuracy and reliability. In this context, successful re-docking (low RMSD) provides support that the used docking setup is able to reproduce the experimental binding mode, strengthening confidence on the grounds that predicted ligand–target interactions work for interpreting experimental bioactivity trends.

### 2.12. Simulation of Molecular Dynamics

Molecular dynamics (MD) simulations were conducted to study the kinetic properties and conformational stability of protein–ligand complexes using GROMACS 5.2 (2020.1) [46]. Ligand topology files were produced by the CGenFF server, and protein parameters were obtained from the CHARMM36 all-atom force field to generate reliable models. The system was neutralized by adding suitable counterions to maintain close to a net charge of 0. All protein–ligand complexes were solvated using the explicit water model TIP3P in a rectangular periodic box to represent the physiological aqueous environment. Before we performed a production run, energy minimization was carried out by the steepest descent algorithm until a maximum force of 1000 kJ/mol/nm (Fmax) was reached in order to avoid contradiction and maintain the structure’s stability. Equilibration was conducted successively following NVT and NPT ensembles to produce thermodynamic stability. Production MD simulations were performed for 100 ns per system to evaluate the dynamic profiles of protein–ligand interactions as a function of time. Trajectory analysis involved RMSD for backbone stability, root-mean-square fluctuation (RMSF) to assess residue-level flexibility, radius of gyration (Rg) to determine protein compactness, and solvent accessible surface area (SASA) analysis plotted against simulation time points in order to represent the degree of surface exposure during the entire simulation period, providing an overall perception of how binding is maintained and conformational fluctuations are taking place at the molecular level as well.

### 2.13. Calculation of MM-PBSA Binding Energy

Free energies for the protein–ligand complexes were calculated using the Molecular Mechanics Poisson–Boltzmann Surface Area (*MM–PBSA*) method [47], which was launched with the g_mmpbsa script program [48]. This approach uses the two main energy terms, which are the molecular mechanics in the gas phase (Δ*EMM*) and the solvation free energy (Δ*Gsolvation*), combined as follows (Equation (5)):

Δ*E* (*MM*–*PBSA*) = Δ*EMM* + Δ*Gsolvation* (5)

The molecular mechanics energy (*EMM*) term consists of the sum of electrostatic interactions (Eele) and van der Waals contributions (EvdW), which correspond to the potential energy of the complex in vacuum. The solvation free energy is separated into polar (Gpol) and non-polar (Gnonpol), where the polar contribution is calculated by solving the Poisson–Boltzmann (PB) equation to consider electrostatic desolvation effects, whereas the non-polar term is obtained from the solvent-accessible surface area (SASA), which describes hydrophobic interactions and cavity formation energy. This computational model affords a thermodynamically informed prediction on ligand binding affinities encompassing both enthalpic and solvation signatures.

### 2.14. Statistical Analysis

All experiments were performed in triplicate, and the results are expressed as mean ± SD (n = 3). Statistical analyses were carried out using GraphPad Prism 9 (v9.5.1; GraphPad Software, San Diego, CA, USA). For comparisons among multiple groups, one-way ANOVA was applied. When all pairwise comparisons between groups were required, Tukey’s multiple-comparison test was used as the post hoc test. When comparisons were restricted to each treatment versus the control group, Dunnett’s multiple-comparison test was used. Differences were considered statistically significant at *p* < 0.05.

## 3. Results

### 3.1. Plant Material Quality Control

The quality assessment of the plant material shown in Table 5 and Table 6 was indicative of *A. cepa* peel’s physicochemical features and safety, which may be exploited for nutraceutical and pharmacological purposes. The material moisture content is 13.78%, which shows a moderate water level, and this should be considered to control the storage time and prevent spoilage. Its acidic pH (3.06) may be perceived as a naturally adverse pH for some parts of microbial flora and also indicates a particular chemical stability of this matrix, having a high content of acidic substances. The ash content (15.21%) further implies the presence of considerable minerals, and the profile is interesting from a nutritional point of view.

Regarding trace metal elements, data on certain plant matrices remain generally limited; in our case, seven elements (Pb, As, Cr, Fe, Ti, Cd, and Sb) were quantified. The measured concentrations for *A. cepa*, As (0.1198 mg/kg), Cd (0.0437 mg/kg), Cr (0.0655 mg/kg), Fe (0.6786 mg/kg), Pb (0.0968 mg/kg), Sb (0.1223 mg/kg), and Ti (0.0729 mg/kg) remain significantly below the maximum limits recommended by FAO/WHO (2009) [49] for As, Cd, Cr, Fe, Pb, and Sb. In general, our results confirm the *A. cepa* peels as a promising material and in compliance with safety regulations for valorization applications in health and nutrition areas.

### 3.2. Phytochemical Tests on A. cepa Peels

The phytochemical analysis of *A. cepa* peels revealed a variety of bioactive secondary metabolites, as listed in Table 7. The abundance of flavonoids (+++), sugar, and holosides (+++) indicated a high concentration of phenolic chemicals and carbohydrates. Gallic tannins (+) were only mentioned as a trace, while sterols, triterpenes (++), and catechic tannins (++) were present in the mean proportion.

On the other hand, in those of anthracenic derivatives, quinone (−), O-heteroside (−), and C-heteroside (−) tests were negative. In a like manner, saponosides (−) were absent. Finally, the screening for alkaloids was positive with Dragendorff (++) and Mayer (++) reagents, and it can be said that this family is present in average amounts.

In general, these findings outline a strong fingerprint towards flavonoids and carbohydrate molecules as a major category, with remarkable sterols/triterpenes and tannins being present in some samples, likely to be suggestive of the demand for *A. cepa* peels for antioxidant and antimicrobial purposes. The nonpresence of anthraquinone derivatives and saponosides associated with the presence of alkaloids underlines a specific profile that could guide future research for discovering and valorizing these classes of compounds in nutraceutical or pharmaceutical preparations.

### 3.3. Phenolic Compound Extraction and Quantitative Analysis

#### 3.3.1. Extraction Yields

**Figure 1:**
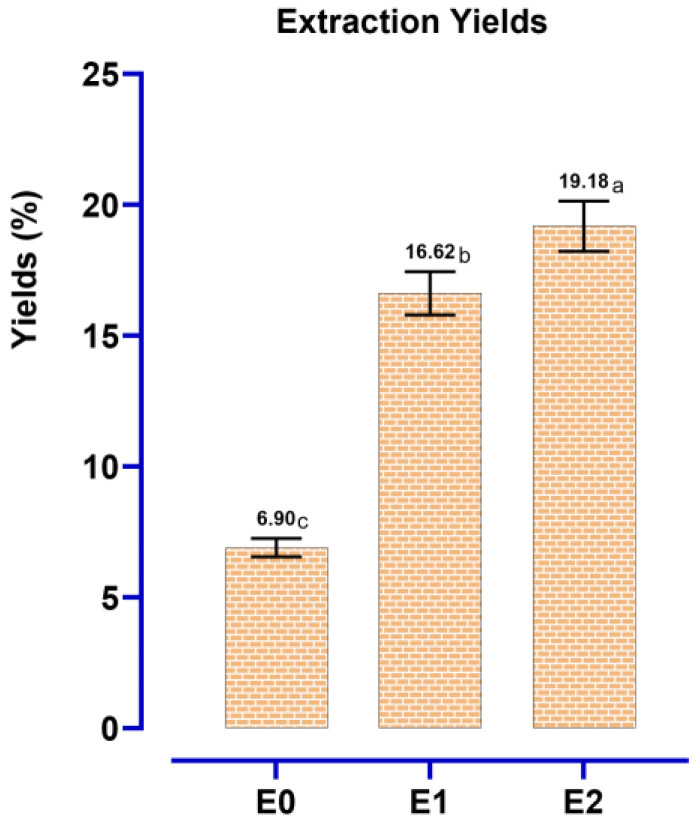
Phenolic compound extraction yields from *A. cepa*: E0: decoction; E1: Soxhlet-obtained aqueous extract; E2: Soxhlet-obtained hydroethanolic extract. Data are presented as mean ± SD (n = 3). Different letters indicate significant differences (*p* < 0.05, one-way ANOVA followed by Tukey’s test).

There were three extractions from *A. cepa* peels: decoction E0, Soxhlet extraction in water E1, and hydroethanolic Soxhlet extraction E2. The results of Figure 1 clearly reflect differences in the yields between different constitutive procedures. The lowest percentage (6.9%) was observed from decoction E0, and the percentages were higher for Soxhlet extractions: 16.62% for E1 and a maximum value of 19.18% for E2.

Overall, the results of this work suggest that for *A. cepa*, Soxhlet extraction (particularly with a hydroethanolic solvent) favors the recovery of phenolics more efficiently than decoction. The better performance of E2 could be attributed to the intermediate polarity of the ethanol–water mixture, which could dissolve a broader range of compounds—from polar ones (some phenolic compounds) to moderately polar—and to the Soxhlet operation, where reflux takes place, enhancing mass transfer. On the other hand, decoction may provide less complete applications of heat to polyphenols and/or partial degradation of some thermolabile molecules, and this could add up to lower yields.

#### 3.3.2. Assessment of Polyphenol, Flavonoid, Condensed Tannin, and Hydrolyzable Tannin Concentrations

Total polyphenol, flavonoid, condensed tannin, and hydrolyzable tannin contents in aqueous extract E0 and Soxhlet extracts (E1 and E2) prepared from *A. cepa* peels were determined spectrophotometrically using standard calibration curves. Gallic acid served as the reference standard for total polyphenol quantification (Y = 0.095X + 0.003; R^2^ = 0.998), quercetin for flavonoid determination (Y = 0.073X − 0.081; R^2^ = 0.995), catechin for condensed tannin estimation (Y = 0.7421X + 0.0318; R^2^ = 0.998), and tannic acid for hydrolyzable tannin measurement (Y = 0.1700X − 0.0006718; R^2^ = 0.996). The results were expressed as milligrams of gallic acid equivalents per gram of extract (mg GAE/g) for polyphenols, milligrams of quercetin equivalents per gram (mg QE/g) for flavonoids, milligrams of catechin equivalents per gram (mg CE/g) for condensed tannins, and milligrams of tannic acid equivalents per gram (mg TAE/g) for hydrolyzable tannins.

**Figure 2:**
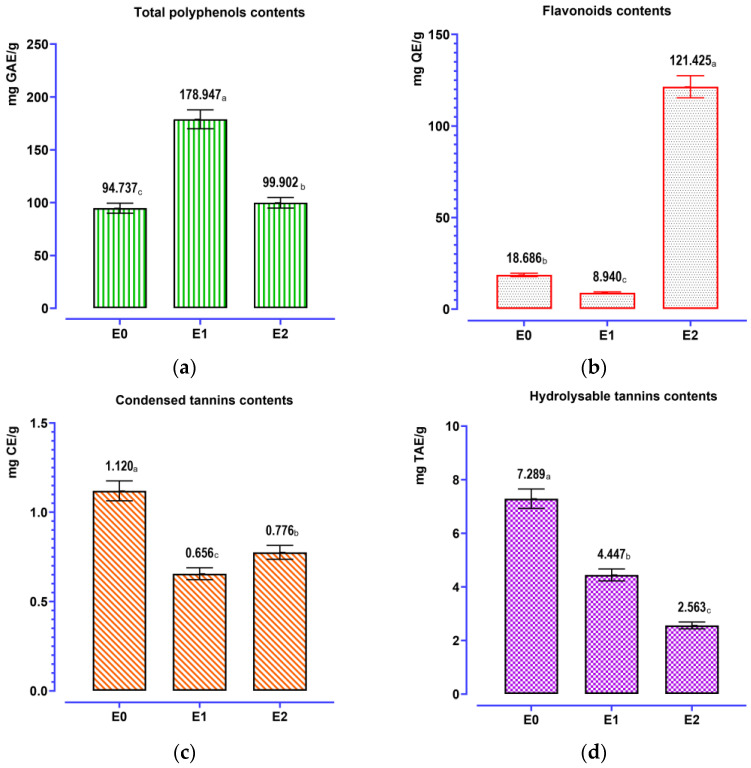
Composition of phenolic compounds: (**a**) total polyphenols; (**b**) flavonoids; (**c**) condensed tannins; (**d**) hydrolyzable tannins. Data are presented as mean ± SD (n = 3). Different letters indicate significant differences among groups (one-way ANOVA followed by Tukey’s multiple-comparison test, *p* < 0.001).

The results of the analysis (Figure 2) highlight a marked difference in content based on the extraction procedure used (*p* < 0.001). The total polyphenol content of the aqueous Soxhlet extract E1 is the highest (178.95 ± 8.95 mg GAE/g), followed by E2 (99.90 ± 4.99 mg GAE/g) and E0 (94.74 ± 4.74 mg GAE/g). On the other hand, the flavonoid amount is highest in hydroethanolic Soxhlet extract E2, which has a remarkable value (121.43 ± 6.08 mg QE/g) that is much higher than E0 (18.69 ± 0.94 mg QE/g) and E1 (8.94 ± 0.45 mg QE/g). This indicates that the hydroethanolic blend is especially suited for the extraction of *A. cepa* flavonoids.

In terms of tannins, the decoction E0 contains the highest contents of both condensed tannin (1.12 ± 0.06 mg CE/g) and hydrolyzable tannin (7.29 ± 0.37 mg TAE/g). Soxhlet extracts produced lower levels: for condensed tannins, E2 (0.78 ± 0.04 mg CE/g) and E1 (0.66 ± 0.03 mg CE/g); for hydrolyzable tannins, E1 (4.45 ± 0.22 mg TAE/g) followed by E2 (2.56 ± 0.13 mg TAE/g).

On the whole, these data have definitively proven that the phenolic profile of *A. cepa* peel extracts was highly affected by the extraction method; E1 favors a high recovery yield of total polyphenols, E2 seems more adequate for extracting flavonoids, and E0 was relatively better for obtaining tannin-enriched (condensed/hydrolyzable) fractions. This distinction is critical to direct the technological choice depending on the objective (flavonoid-rich antioxidants or tannic fractions).

#### 3.3.3. Analysis and Identification of Polyphenols in A. cepa Extract by High-Pressure Liquid Chromatography–Mass Spectrometry (HPLC/UV-ESI-MS)

**Figure 3:**
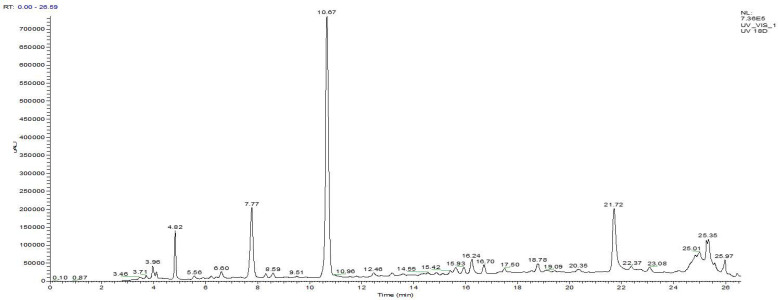
HPLC chromatograms of compounds extracted from the decocted *A. cepa*.

The analysis of the decoction of *A. cepa* peels was performed by HPLC/UV ESI MS, and the corresponding chromatogram is presented in Figure 3.

By comparing retention times with mass spectra in the negative mode, a total of 20 compounds were identified (Table 8). The molecules were tentatively identified on the basis of pseudomolecular ions [M–H]^−^ and characteristic fragments. Similarly, class distribution (Table 9) depicts obvious dominance of flavonoids (84.93% of total area), followed by phenolic acids (9.45%), phenolic compounds (2.84%), polyphenols (2.20%), and a minute portion of saponins (0.58%), reaffirming the abundance of bioactive secondary metabolites in *A. cepa* peels.

Isorhamnetin (RT 10.67 min) is the major marker of the extract (30.26%). It generates the [M–H]^−^ ion at *m*/*z* 315 and fragmentation at *m*/*z* 300, 271, 255 and 151. Quercetin (RT 25.01 min; 14.03%) was detected with [M–H]^−^ at *m*/*z* 301, and further fragments were detected at *m*/*z* 179, 151, 273, and 271. There are many glycosylated flavonoid derivatives, including quercetin 4′-*O*-glucoside (RT: 21.72 min; 12.42%), but it is not exclusive, and it was observed with [M–H]^−^ at *m*/*z* 463 and a characteristic fragment at *m*/*z* 301 arising from the loss of sugar residues. Other examples are hyperoside (RT 25.35 min; 4.61%) and quercetin 3-*O*-xyloside (RT 25.59 min; 2.28%), whereby they showed identical fragmentation patterns to those of glycosylated flavonols. An anthocyanoside, cyanidin 3-*O*-glucoside (RT 7.77 min; 8.41%), was confirmed by [M–H]^−^ at *m*/*z* 483, and fragments were confirmed at *m*/*z* 303, 241, and 285. Other flavonoids and flavan-3-ol oligomers were identified, such as type B procyanidin (RT 22.37 min; 3.89%), (−)-epicatechin (RT 16.24 min; 2.45%), patuletin (RT 23.08 min; 4.01%), and glabrol (RT 24.21 min; 1.98%), highlighting the structural diversity of the flavonoid fraction.

**Figure 4:**
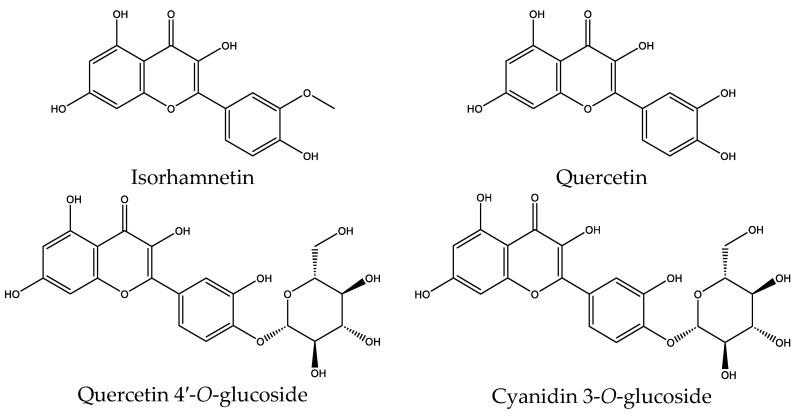
Configuration of the principal compounds.

In parallel, several phenolic acids were detected, notably gallic acid (RT 3.46 min; 0.65%) with [M–H]^−^ at *m*/*z* 169 and a fragment at *m*/*z* 125 compatible with decarboxylation; chlorogenic acid (RT 6.60 min; 0.87%) with [M–H]^−^ at *m*/*z* 353 and fragments at *m*/*z* 191, 179, 173, and 135; and ferulic acid (RT 3.96 min; 1.29%) identified by [M–H]^−^ at *m*/*z* 193 and fragments at *m*/*z* 178, 149, and 134. Ellagic acid (RT 25.97 min; 3.00%) was identified by [M–H]^−^ at *m*/*z* 301, and fragments were identified at *m*/*z* 283, 257, 229, and 185; other derivatives, gallic acid hexoside (RT 18.78 min; 2.85%) and gentisic acid (RT 15.62 min; 0.79%), complete this profile. Finally, the minor non-flavonoid fraction includes trans-resveratrol (RT 20.35 min; 2.20%), detected at *m*/*z* 227 with fragments at *m*/*z* 185, 159, and 143, and a saponin, Alliospiroside A (RT 15.93 min; 0.58%) presenting [M–H]^−^ at *m*/*z* 707 and fragments at *m*/*z* 563, 545, and 401. Figure 4 illustrates the structures of the main identified compounds, notably isorhamnetin, quercetin, quercetin 4′ O glucoside, and cyanidin 3 O glucoside, reinforcing the chemical interpretation and putting into perspective the potential biological activities associated with the decoction of *A. cepa* peels.

### 3.4. Antioxidant Activity

**Figure 5:**
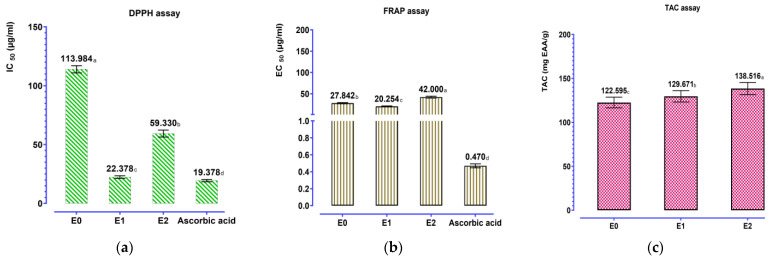
Antioxidant efficacy of ascorbic acid and extracts as determined by (**a**) DPPH, (**b**) FRAP, and (**c**) TAC tests. Data are presented as mean ± SD (n = 3). Different letters indicate significant differences among groups (one-way ANOVA followed by Tukey’s multiple-comparison test, *p* < 0.001).

The antioxidant potential of *A. cepa* peel extracts was evaluated using three complementary in vitro assays: 2,2-diphenyl-1-picrylhydrazyl (DPPH) radical scavenging activity, ferric reducing antioxidant power (FRAP), and total antioxidant capacity (TAC). Three extracts were evaluated: aqueous decoction extract E0, Soxhlet water extract E1, and Soxhlet hydroethanolic extract (E2, ethanol/water 70:30 *v*/*v*). Ascorbic acid was used as the reference standard for all assays, and a calibration curve was prepared: Y = 1.013X − 8.032; R^2^ = 0.9893 (DPPH) and Y = 0.004760X + 0.09740; R^2^ = 0.8963 (FRAP) and Y = 0.04066X + 0.02110; and R^2^ = 0.9949 (TAC). The values (Figure 5) demonstrate that the extracts showed strong antioxidant capacity: There was a high variability associated with the extraction procedure, and it was deemed significant (*p* < 0.001). In the DPPH test extract, E1 was the most active among the extracts with an IC_50_ = 22.34 ± 1.12 µg/mL, followed by E2 (59.33 ± 2.97 µg/mL) and E0 (113.98 ± 3.06 µg/mL), whereas ascorbic acid presented an IC_50_ of 19.38 ± 0.97 µg/mL. Similarly, the FRAP assay was consistent, with E1 being the most active (EC_50_ = 20.25 ± 1.01 µg/mL), followed by E0 (27.84 ± 1.39 µg/mL) and then E2 (42.00 ± 2.10 µg/mL), but ascorbic acid was still considerably superior in activity (0.47 ± 0.02 mg/g). On the contrary, the TAC assay showed an opposite tendency, showing E2 with the maximum total antioxidant capacity (138.52 ± 6.93 mg AAE/g), followed by E1 (129.67 ± 6.48 mg AAE/g) and finally E0 (122.60 ± 6.13 mg AAE/g). All these findings point out that the extraction procedure is a key factor affecting antioxidant activity expression and confirm the interest of a multi-test methodology since DPPH/FRAP assays (radical scavenging/reducing power) and TAC (overall capacity) response may represent partially different antioxidant system actions.

### 3.5. Antimicrobial Activity

The decoction of *A. cepa* peels showed considerable antibacterial effectiveness against a wide spectrum of Gram-positive bacteria (GPC) and Gram-negative bacteria (GNB), as well as yeasts and molds, as indicated by the MIC, MBC, and MFC results presented in Table 10. In general, extracts exhibit lower antibacterial/antifungal activity when compared with the reference antibiotics/antifungals, but what is interesting is a distinct inhibition of selected microorganisms. In GPC, the activity varies from sensitive for *Staphylococcus epidermidis* and *S. agalactiae* (MIC = 150) to moderate sensitivity in *S. aureus* producing β-lactamase (MIC = 300). On the contrary, lowered sensitivity profiles are observed against the MRSA isolate (*S. aureus* STAIML/MRS/mecA/HLMUP/BLACT) (MIC = 2500 µg/mL) and with respect to some streptococci (group D: MIC = 2500 µg/mL, *S. acidominimus*: MIC = 600 µg/mL; *S. porcinus*: MIC = 1200 µ/mL). *Enterococci* (*E. faecalis* and *E. faecium*) are less sensitive (MIC being 5000 µg/mL for both enterococcal species; MBC is also 5000 µg/mL), reflected by showing relatively less activity against these two species.

In GNB, the extract shows particularly marked inhibitions against certain enterobacteria and *Pseudomonas*, with the lowest MICs observed for *E. cloacae* and *E. cloacae* 2280 (MIC = 75 µg/mL; MBC = 150 µg/mL) and *P. aeruginosa* (MIC = 75 µg/mL), as well as *P. aeruginosa* 1124 (MIC = 150 µg/mL). Interesting activity is also noted against *K. pneumoniae* (MIC = 300 µg/mL), *K. pneumoniae* 1015 (MIC = 600 µg/mL; MBC = 600 µg/mL), *P. mirabilis* (MIC = 300 µg/mL; MBC = 600 µg/mL), and *S. marcescens* (MIC = 300 µg/mL; MBC = 600 µg/mL), as well as against *E. coli* and ESBL-producing *E. coli* (MIC = 600 µg/mL; MBC = 1200 µg/mL) and ESBL-producing *E. coli* isolate 5765 (MIC = 1200 µg/mL), indicating sustained inhibition despite the presence of resistance mechanisms. Additional strains show variable sensitivity, such as Enterobacter aerogenes (MIC = 1200 µg/mL; MBC = 2500 µg/mL), *P. fluorescens* (MIC = 600 µg/mL), *P. putida* (MIC = 2500 µg/mL), *C. koseri* (MIC = 2500 µg/mL), and *Y. enterocolitica* (MIC = 1200 µg/mL). On the other hand, lower sensitivity is observed for *A. baumannii* and *A. baumannii* 2410 (MIC = 2500 µg/mL) and for several enteric pathogens such as *Salmonella* sp. and *Shigella* sp. (MIC = 2500 µg/mL; MBC = 2500 µg/mL).

In GNB, the extract generally displays stronger inhibitions of some enterobacteria and *Pseudomonas*, and the lowest MICs are against *E. cloacae* (MIC = 75 µg/mL), *E. cloacae* 2280 (MIC = 75 µg/mL), and *P. aeruginosa* (MIC = 75 µg/mL), as well as *P. aeruginosa* 1124 (MIC = 150 µg/mL; MBC = 150 µg/mL). Moderate activity is also observed against *K. pneumoniae* (MIC = 300 µg/mL), *K. pneumoniae* 1015 (MIC = 600 µg/mL), *P. mirabilis* (MIC = 300 µg/mL; MBC = 600 µg/mL), and *S. marcescens* (MIC = 300 µg/mL; MBC = 600 µg/mL), as well as against *E. coli* and ESBL-producing *E. coli* (MIC = 600 µg/mL) and ESBL-producing *E. coli* isolate 5765 (MIC = 1200 µg/mL), indicating sustained inhibition despite the presence of resistance mechanisms. Additional strains show variable sensitivity, such as *E. aerogenes* (MIC = 1200 µg/mL), *P. fluorescens* (MIC = 600 µg/mL), *P. putida* (MIC = 2500 µg/mL), *C. koseri* (MIC = 2500 µg/mL), and *Y. enterocolitica* (MIC = 1200 µg/mL). However, lower sensitivity values were found for *A. baumannii* and *A. baumannii* 2410 (MIC = 2500 µg/mL) and some enteric pathogens such as *Salmonella* sp. and *Shigella* sp. (MIC = 2500 μg/mL).

At the level of fungi, antifungal activity seems generally weak to moderate; most *Candida* species were found to have high MICs, with MFCs of generally >5000 µg/mL (*C. albicans*: MIC = 5000 µg/mL; MFC > 5000 µg/mL; *C. kefyr*: MIC = 5000 µg/mL; MFC > 5000 µg/mL; *C. krusei*: MIC > 5000 µg/mL; MFC > 5000 µg/mL; *C. parapsilosis*: MIC = 5000 µg/mL; MFC > 5000 µg/mL; *C. tropicalis*: MIC > 5000 µg/mL; MFC > 5000 µg/mL; *C. dubliniensis*: MIC > 5000 µg/mL; MFC > 5000 µg/mL), indicating limited inhibition at the tested concentrations. *S. cerevisiae* has moderate sensitivity (MIC = 1200 µg/mL), and it is poorly sensitive to *A. niger* (MIC = 5000 µg/mL). As anticipated, all antibiotics (gentamicin, amoxicillin–clavulanic acid, vancomycin, trimethoprim–sulfamethoxazole, and penicillin G) and the antifungal standard terbinafine have much lower MIC values than those of the extract, indicating higher intrinsic potency.

However, the recorded activity of *A. cepa* decoction (especially against *E. cloacae* and *P. aeruginosa*) indicates specific biologically active compounds, which may have contributed to the antimicrobial effects, and calls for future studies aimed at the isolation and identification of active constituents, as well as elucidating mechanistic aspects otherwise; this would provide a better understanding of the potential for using it as co-adjuvant or even as a source of new antimicrobials.

### 3.6. Anticoagulant Activity

**Figure 6:**
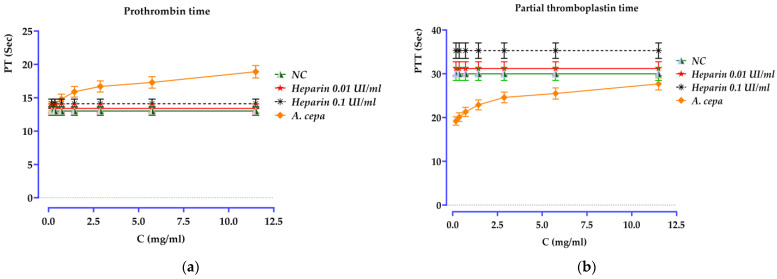
Impact of *A. cepa* decocted extract E0, standard control (NC), and heparin on prothrombin time (**a**) and partial thromboplastin time (**b**).

The prothrombin time (PT) and activated partial thromboplastin time (aPTT) tests, significant indicators of hemostasis, were employed to assess the anticoagulant efficacy of the *A. cepa* peel decoction (E0) (Figure 6). Compared to the negative control (NC), the extract induced a significant dose-dependent modification of coagulation times (*p* < 0.001), reflecting an overall increasing anticoagulant effect with the tested concentration (0.179–11.5 mg/mL). For the PT, a progressive prolongation was observed, increasing from 13 s (NC) to 18.9 s at 11.5 mg/mL, whereas heparin, used as a reference, only caused slight variations under the test conditions (13.4 s at 0.01 IU/mL and 14.1 s at 0.1 IU/mL). Similarly, the PTT gradually increased with the extract, from 19.2 s to 27.7 s between 0.179 and 11.5 mg/mL, while the values obtained with heparin were higher but remained relatively stable (31.2 s at 0.01 IU/mL and 35.3 s at 0.1 IU/mL), and the NC remained at 30 s. Together, these results indicate that the decoction of *A. cepa* exerts a moderate but concentration-dependent anticoagulant effect, suggesting a potential interaction with components of the coagulation pathways. Although the underlying molecular mechanisms remain to be clarified, these data support the interest in continuing investigations to identify the responsible constituents, elucidate the involved biological targets, and evaluate the safety profile of the extract.

The prothrombin time (PT) and activated partial thromboplastin time (aPTT), two of the most important parameters for both intrinsic and extrinsic coagulation, were employed to assess the anticoagulant activities of the *A. cepa* peel decoction (E0) (Figure 6). With respect to the negative control (NC), a marked dose-dependent alteration of coagulation times (*p* < 0.001) was induced by the extract, with an overall trend toward a higher anticoagulant effect at the tested systemic concentration interval (0.179–11.5 mg/mL). Regarding the PT, a progressive extension could be observed, varying from 13 s (NC) to 18.9 s at the highest *A. cepa* concentration employed for testing (11.5 mg/mL), while heparin used as a reference only induced minimal changes under similar test conditions (13.4 and 14.1 s at 0.01 IU/mL and 0.1 IU/mL, respectively). Similarly, the PTT increased in a concentration-dependent manner with the extract (from 19.2 s to 27.7 s between 0.179 and 11.5 mg/mL), but higher values were also obtained with heparin, which was, however, relatively stable (31.2 s at 0.01 IU/mL and around 35–36 s from then on), whereas NC remained around the time value of each test, i.e., at about 30 s. These data first illustrate that the *A. cepa* decoction exhibits moderate but significant anticoagulant activity and secondly suggest a possible interaction with some coagulation pathway components. Despite the unresolved molecular mechanisms, these findings justify further studies aimed at identifying the active principles and the affected biological targets and evaluating the safety of the extract.

### 3.7. Antidiabetic Activity

#### 3.7.1. Assessment of the Inhibitory Impact of Decocted Extract on the Activity of α-Amylase and α-Glucosidase In Vitro

**Figure 7:**
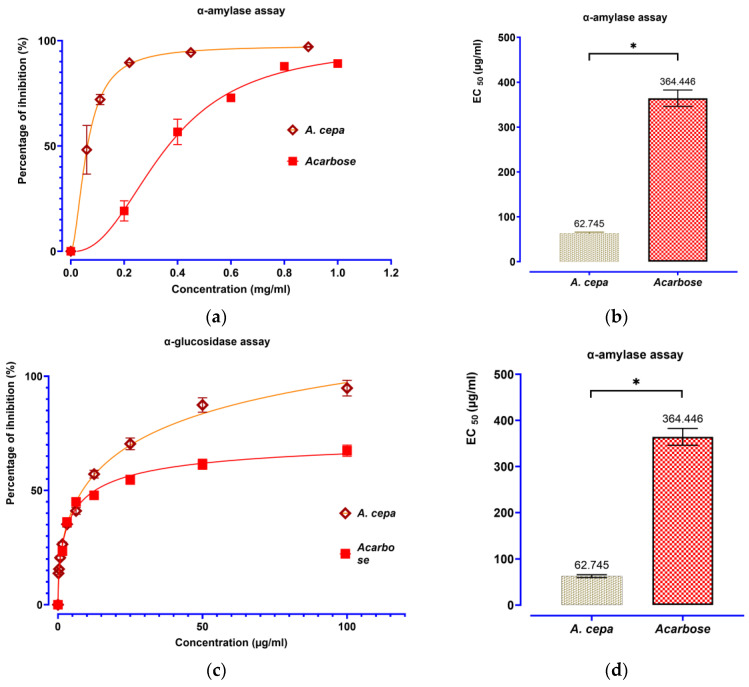
Percent inhibition and EC_50_ of the inhibitory effects on α-amylase (**a**,**b**) and α-glucosidase (**c**,**d**) activities by *A. cepa* decocted extract compared with acarbose (positive control) in vitro. Data are presented as mean ± SD (n = 3). Statistical analysis was performed using an unpaired two-tailed *t*-test. *p* < 0.05 (*).

The decoction extract of *A. cepa* peels E0 was tested in vitro for its antidiabetic activity to establish its clinical importance through inhibiting the carbohydrate hydrolyzing activity of two enzymes, α-amylase and α-glucosidase, as shown in Figure 7. An inhibitory effect of α-amylase was dose-dependent, and at 0.89 mg/mL, it lowered enzyme activity by 96.98%, which is similar to that (97.21%) observed at 0.89 mg/mL for acarbose as well. The suppressor activity of the extract is not as potent as acarbose at high doses in terms of the analysis of inhibitory potency, whereas at smaller doses, the data reveal that the EC_50_ value (62.745 ± 3.137 µg/mL) was significantly lower than that of acarbose (364.446 ± 18.2 µg/mL), indicating higher action efficiency per unit concentration. The extract also showed a concentration-dependent inhibition of α-glucosidase, with maximum inhibitory activity of 92.37% at 100 mg/mL when compared with acarbose at 97.23%. The EC_50_ of the extract (8.489 ± 0.424 µg/mL) was also less than that of acarbose (17.269 ± 0.863 µg/mL), which exhibited stronger inhibitory potency. Taken together, these findings suggest the antidiabetic potential of *A. cepa* extracts derived from different parts of the plant and support its interest as an interesting source for natural inhibitors in the modulation of postprandial plasma glucose; however, the identification of responsible compounds and investigation of mechanisms (affinity, inhibiting dynamics, and in vivo validation) are required to validate this hypothesis.

#### 3.7.2. Acute Taxicity Study

The acute toxicity study of the decocted extract of *A. cepa* showed it to be non-toxic up to the 2 g/kg dose level. During the study time, no side effects (diarrhoea, vomiting, abnormal mobility, and mortality) after oral administration of the extract were reported. This observation implies a high safety margin and thus a potential therapeutic value of *A. cepa* without immediate toxicological effects.

#### 3.7.3. Investigation of the Antihyperglycemic Efficacy of *A. cepa* Decocted Extract in Normal Rats In Vivo

**Figure 8:**
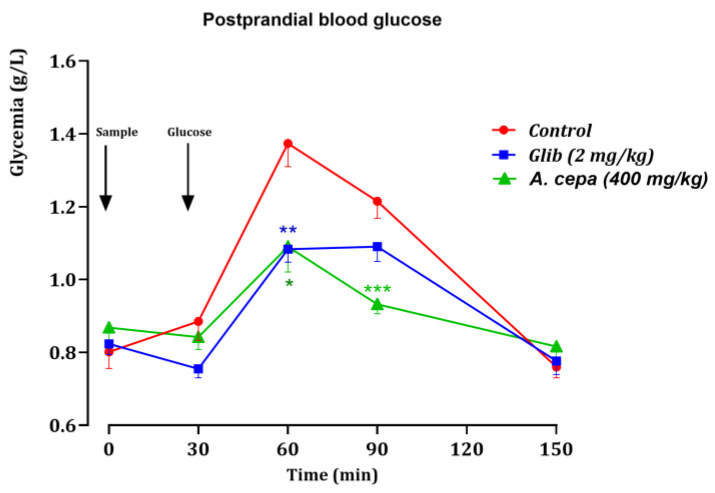
Postprandial blood glucose levels in normal rats following administration of the test products (*A. cepa* decocted extract, 400 mg/kg, and glibenclamide, 2 mg/kg). Data are presented as mean ± SD (n = 6). Statistical analysis was performed using one-way ANOVA followed by Dunnett’s post hoc test. *p* < 0.05 (*), *p* < 0.01 (**), and *p* < 0.001 (***).

**Figure 9:**
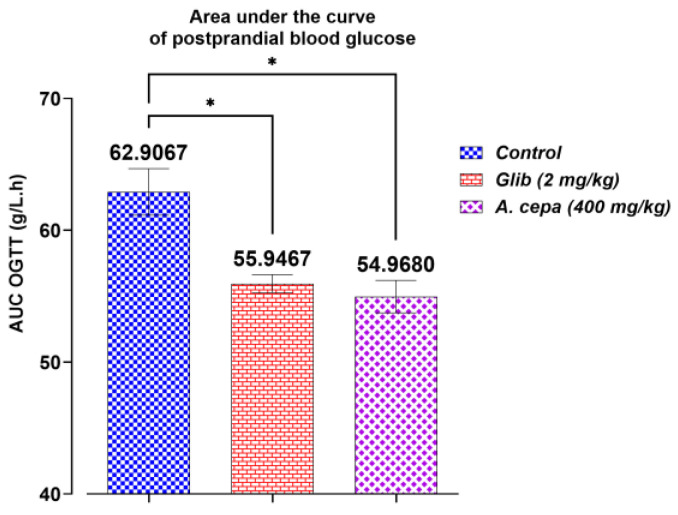
Area under the curve (AUC) of postprandial blood glucose during OGTT in normal rats after treatment with glibenclamide (2 mg/kg) or *A. cepa* decocted extract (400 mg/kg). Data are shown as mean ± SD (n = 6). Statistical analysis: one-way ANOVA followed by Dunnett’s post hoc test vs. control. *p* < 0.05 (*).

The antihyperglycemic effect of the *A. cepa* decoction (400 mg/kg) was investigated in normal rats employing the oral glucose tolerance test (OGTT). The evolution of postprandial blood glucose over 150 min and the AUC comparing treatments are presented in Figure 8 and Figure 9 showing which treatments were able to limit the glycemic peak and accelerate the return to basal values.

- Test of oral glucose tolerance

After glucose overload, the control group (distilled water) showed a rapid increase in blood glucose levels, with a clear peak at 60 min. In this group, the values at 60 min generally ranged around 1.16–1.57 g/L (Figure 8), indicating a marked postprandial hyperglycemia, followed by a gradual decrease at 90 min and then 150 min.

Conversely, glibenclamide (2 mg/kg) significantly attenuated this elevation: at 60 min, blood glucose levels remained generally lower (around 1.01–1.17 g/L), confirming the expected effect of the reference treatment on post-load hyperglycemia control.

Consistently, the decoction of *A. cepa* (400 mg/kg) also reduced the postprandial increase compared to the control, with values at 60 min generally lower than those of the control group and a tendency toward a faster return to levels close to the initial blood glucose at later time points (90 and 150 min). All data indicate significant differences between the treated groups and the control (*p* < 0.01), showing that the extract exerts a protective effect against the glycemic peak induced by the glucose load.

These observations support a postprandial antihyperglycemic effect of the *A. cepa* decoction, comparable in general dynamics to a reference antidiabetic treatment (glibenclamide), although the exact’s intensity depends on the time points and inter-individual dispersion. This action is consistent with previous in vitro results showing the inhibition of α amylase and α glucosidase enzymes, mechanisms likely to slow down the release/absorption of glucose after ingestion.

- Areas under the curve (AUCs) of postprandial glucose concentrations

The areas under the curve (AUCs) of postprandial blood glucose (Figure 9) are shown. The analysis of areas under the curve showed marked variations in three experimental groups (control; glibenclamide 2 mg/kg; *A. cepa* decoction 400 mg/kg). The controls have the largest glycemic exposure, which varies between 59.72 and 71.28 g/L·h, with an average of about 62.9067 g/L·h. Glibenclamide reduces AUC much more (individual values lie between 53.36 and 57.72 g/L·h; average = 55.9467 g/L·h). The decoction of *A. cepa* also exhibits a substantial decrement in AUC when compared to the control (available values 52.2–59.6 g/L·h; mean = 54.9680 g/L·h), representing an attenuation of glucose peaks and/or faster normalization of blood glucose following saturation with glucose load/dose. These data support the existence of an antihyperglycemic postprandial effect of *A. cepa*, which is as powerful as that of glibenclamide in our laboratory (*p* < 0.05 vs. control) and further emphasize interest in the extract as a phytotherapeutic alternative for preventing postprandial hyperglycemia.

### 3.8. Molecular Docking

The molecular docking pattern of the four major flavonoids in the decoction (isorhamnetin, quercetin, quercetin 4′-*O*-glucoside, and cyanidin 3-*O*-glucoside) shows that they have good binding affinities with respect to antibacterial, antifungal, antioxidant, and antidiabetic targets, as well as anticoagulate targets (Table 11 and Table 12). The antibacterial score is prominently high in the case of isorhamnetin on 3IX3 (−9.9 kcal/mol) and quercetin at 4FS3 (−9.8 kcal/mol), which depict polar anchoring through Ala97. Ser197 is strengthened by π–π interactions with Phe96 and π–alkyl with Ile94/Arg40, thus obstructing fatty acid production found in Staphylococcus aureus by inhibiting FabI–NADPH binding. For antifungal action, quercetin 4′-*O*-glucoside on 5FSA (−9.7 kcal/mol) and cyanidin 3-*O*-glucoside on 5V5Z (−9.0 kcal/mol) form hydrogen bonds with Ser378, Gly301, and Tyr505, supported by nonpolar contacts with Leu376, Met508, and Phe228, ensuring CYP51 blockage to interrupt the sterol biosynthetic pathway. A π–anion interaction with Asp282 is involved in NAD(P)H oxidase and reactive species generation. The antioxidant activity shows higher affinity, requiring a lower reducing potential for quercetin 4′-*O*-glucoside on 2CDU (−10.5 kcal/mol), featuring multiple hydrogen bonds (with Thr97, Gly114, Ser115, and Ser41). The antidiabetic action is based on inhibition of α-amylase (4W93, −8.2 kcal/mol for quercetin 4′-*O*-glucoside) and hydrogen bonding to catalytic residues Asp197/Glu233, and this is stabilized by π–π (Tyr62) and π–cation (Trp59) interactions, as they slow down starch digestion [50]. Lastly, anticoagulant activity upon thrombin (4UFD, −8.5 to −8.7 kcal/mol) is due to a salt bridge interaction with Asp58/Asp17 and hydrogen bonded network (Asp73, Thr163, Glu174, and Arg204), which inhibits fibrin formation. These findings imply a polypharmacological synergy involving multiple targets, which is presumably dominated by quercetin 4′-*O*-glucoside; however, these predictions remain to be verified experimentally in vitro or in vivo, with the aim of establishing functional side effect profiles due to these interactions.

### 3.9. MD Simulations

#### 3.9.1. RMSD and RMSF Analysis

**Figure 10:**
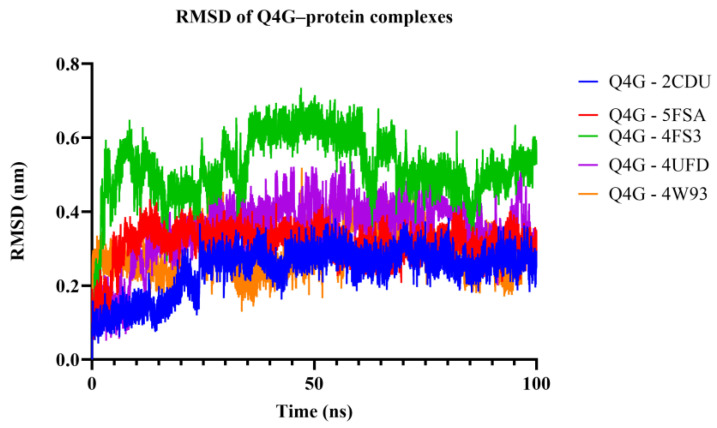
Comparative root mean square deviation (RMSD) analysis of Quercetin 4′-*O*-glucoside (Q4G) in complex with the different proteins studied.

In order to assess the conformational stability of the studied Quercetin 4′-*O*-glucoside (Q4G)-protein complexes, we followed its temporal evolution through RMSD (root-mean-square deviation) calculation of the protein backbone during 100 ns molecular dynamics simulations. All systems (Figure 10) show this initial phase of adjusting with different degrees of stabilization. The Q4G-4W93 and Q4G-2CDU complexes that display the lowest RMSD values also exhibit relatively stable plateaus during production around 0.20–0.30 nm and 0.22–0.32 nm, reflecting a more overall steady skeletal organization of the binding modes. In contrast, the Q4G-5FSA and Q4G-4UFD systems have intermediate RMS deviation values (between 0.30 and 0.40 nm or between 0.35 and 0.50 nm) that correspond to the high structural variability of its C-terminus. In particular, the Q4G-4FS3 complex shows the largest RMSD values (0.45–0.70 nm) and larger fluctuations, which are consistent with more remarkable conformational rearrangements in the course of the simulation. The RMSD profiles altogether indicate, under the simulated conditions, relatively less stable graphene–Q4G complexes compared to those of 4W93 and 2CDU, with the highest skeletal deviations shown for 4FS3.

**Figure 11:**
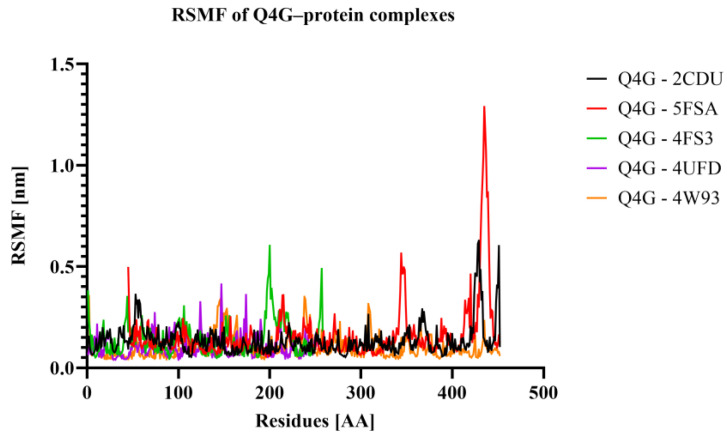
Comparative root mean square fluctuation (RMSF) analysis of the backbone atoms of Q4G and its complexes.

The RMSF (root mean square fluctuation) was determined in order to analyze the residual flexibility of amino acids during MD simulations. Regions with high RMSF are typically more mobile, usually corresponding to loops or termini; those with low values are rigid and maintain stable local conformations. As can be seen in Figure 11, the majority of Q4G–protein complexes display low fluctuations for all residues, indicating that, on the whole, the structure is retained throughout the simulation. However, distinct differences are observed at certain local maxima. The flanking region of Q4G-5FSA, the most remarkable peak of fluctuations (1.2–1.3 nm), is observed for the Q4G-5FSA complex in the 420–440 interval, which indicates this part’s high flexibility. Fewer intensity bands are further detected for Q4G-2CDU at another region (410–440, 0.5–0.6 nm), but that of Q4G-4FS3 has a clear peak at around 180–220 as well. On the other hand, Q4G-4W93 shows a flatter and less varying profile. Overall, these data suggest that although the overall stability of some complexes (Q4G-5FSA in particular) remains relatively preserved, locally, more flexible regions are present that may correspond to specific conformational adaptations throughout the trajectory.

#### 3.9.2. Radius of Gyration (Rg) Analysis

**Figure 12:**
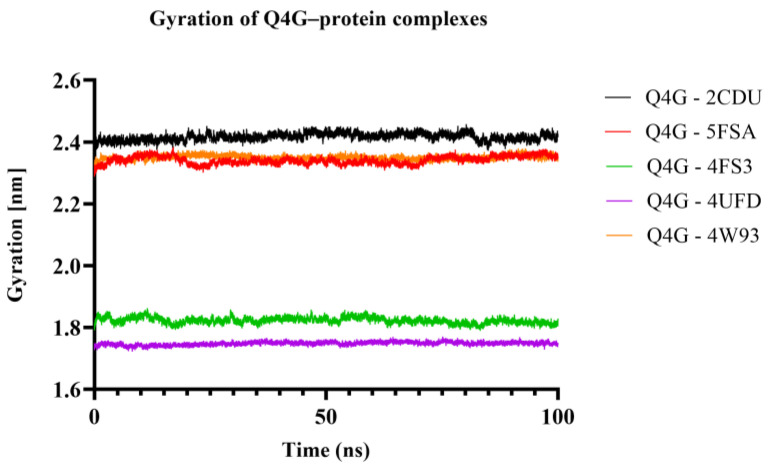
Comparative radius of gyration Rg profiles for each system over 100 ns of simulation.

The radius of gyration (Rg) was used to estimate the overall compactness of the Q4G-protein complexes throughout molecular dynamics simulations. As shown in Figure 12, all systems present relatively constant Rg values, with small-amplitude fluctuations around a plateau value of 60–70, indicating a lack of significant changes in compactness (unfolding/structural collapse) throughout the simulation. The Rg values are specific for the target, and they correspond mainly to differences in the size and architecture of studied proteins. In general, the preservation of an approximately constant Rg indicates that the presence of Q4G does not cause a dramatic global rearrangement in the structure and that complexes remain stable at least within the simulated environment.

#### 3.9.3. Solvent-Accessible Surface Area (SASA) Analysis

**Figure 13:**
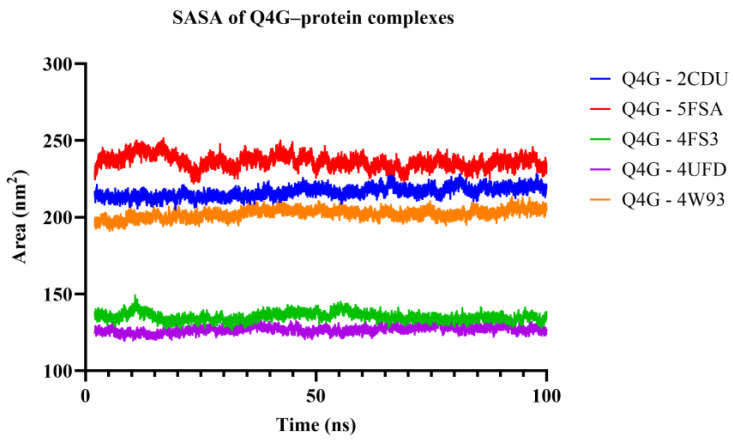
Comparative solvent-accessible surface area (SASA) profiles for each system over 100 ns of simulation.

The SASA of the protein was calculated to monitor the dynamic profile of protein solvent exposure during molecular dynamics simulation. As can be seen in Figure 13, the fluctuations for the SASA profiles of the Q4G–protein complexes present anintermediate degree of fluctuation around average values with stable enough averages, and no large drift is detected, indicating that there were no major global changes in the complex structure during simulations. Overall, the absolute SASA values differ among the targets but mainly result from inherent differences in size and organization of the studied proteins rather than from a ligand-specific effect. In general, the establishment of a quasi-steady state of SASA confirms that the conformational stability of the systems in the presence of Q4G is maintained under simulated conditions.

#### 3.9.4. Binding Free Energy Calculations

The binding free energies of the Q4G-protein complexes were estimated using the Molecular Mechanics Poisson–Boltzmann Surface Area (MM-PBSA) method (Table 13) in order to compare the thermodynamic favorability of the interaction. Overall, the gas-phase contributions (ΔG__GAS_), dominated by van der Waals (ΔE__VDW_) and electrostatic (ΔE__EEL_) terms, are favorable for all systems, while polar solvation (ΔE__PB_) constitutes the main unfavorable contribution, which is partially compensated by the non-polar solvation term (ΔE__NPOLAR_). Among the complexes studied, Q4G-2CDU exhibits the most favorable total binding energy (ΔG__TOTAL_ = −69.77 kcal·mol^−1^), driven by particularly strong van der Waals interactions (ΔE__VDW_ = −81.66 kcal·mol^−1^) and a very favorable ΔG__GAS_ (−130.06 kcal·mol^−1^). The other systems show significantly less negative ΔG__TOTAL_ values (from −24.67 to −10.55 kcal·mol^−1^), indicating a lower predicted affinity, particularly when the polar solvation penalty (ΔE__PB_) increases sharply, as observed for 4UFD and 4W93. Overall, these results suggest a preferential interaction of Q4G with 2CDU according to MM-PBSA, consistent with a more favorable non-covalent “packing” contribution.

## 4. Discussion

The data reported on *A. cepa* peels are in support of the safety and richness of plant matrices for prospective nutraceutical and pharmacological applications. Overall, our quality control, phytochemical profiling (HPLC/ESI-MS), bioassays, and in silico analyses all together provide us with a coherent interpretation; onion peels are characterized by a polyphenol-rich, apparently safe matrix with multi-mechanistic bioactivities that may favor nutraceutical/pharmacological valorization, which is in line with the recent literature suggesting the value of onion peels as agro-food co-products [6,51].

The results of the physicochemical properties indicate that this material could be used as an industrial raw ingredient. Their moisture content is relatively low and lies well within the optimal range for medicinal plants intended for formulation, as it has not been observed to compromise product stability and conservation [52,53], yet they should be properly stored to avoid microbial growth and residual enzymatic activity. The low pH suggests an organic acid- and phenolic compound-rich substrate, which may assist polyphenol solubilisation/extraction and partially mitigate microbial loads, as observed by Samota et al. [54], Rodríguez Galdón et al. [55], and Gil-Martín et al. [56]. The ash value additionally implies possibly high mineral contents, a favorable characteristic in the nutritional valorization of plant by-products [57], but this needs to be verified through elemental profiling.

Safety-wise, regulated metals stayed below FAO/WHO limits (2009) so as to reassure low toxicological concern in the sourcing modes studied. Nonetheless, it has been observed that geographic and agronomic variability might result in different metal loads; thus, traceability and monitoring per batch are pivotal before any scaling up is done [58,59,60].

Phytochemical screening indicates the presence of a high quantity of flavonoids and sugar/holoside, whereas sterols/triterpene and catechin tannin also have some contributions. This pattern is typical of peripheral plant tissues, where polyphenols, such as flavonols, accumulate at high levels for protection against oxidative stress and pathogen attack [61,62,63]. The simultaneous presence of alkaloids can at least partly explain some activities, specifically antimicrobial activities, but the exact contribution depends on the identity and bioavailability of the compounds [64]. The lack of saponins and anthraquinones mechanistically suggests that polyphenols are probably responsible for a large part of antidiabetic bioactivity. These are extensively associated with antioxidant effects via membrane alteration/enzymatic inhibition [64,65,66]. Moreover, the developed enzyme–ligand interactions are important for some activities that prove to be antidiabetic and anti-coagulant, primarily due to tannins’ affinity for proteins and their ability to inhibit digestive enzymes [67,68].

Extraction yields (E0 < E1 < E2) highlight the strong influence of the extraction mode. The higher recovery obtained with hydroethanolic Soxhlet extraction is consistent with the literature showing that ethanol–water mixtures solubilize a broader range of phenolics and that continuous reflux enhances mass transfer and extraction efficiency [69,70]. In contrast, the lower yields observed for decoction agree with reports attributing reduced recovery to thermal degradation of thermolabile compounds and the weaker ability of water-only systems to solubilize less hydrophilic metabolites [71,72]. Accordingly, our results corroborate previous studies reporting the overall superiority of hydroethanolic and Soxhlet-type procedures over aqueous extraction for polyphenol recovery [73].

The available literature for *A. cepa* suggests that the amount of phenolic compounds can differ significantly depending on the analyzed organ, and comparisons with studies carried out on intact bulbs or internal tissues may therefore be imperfect; the external fractions (peels) are generally more concentrated in phenolic metabolites than the internal tissues. In the literature, total polyphenols are very variable according to varieties and protocols: from 8.05 to 10.8 mg GAE/100 g [74] and 84.5 mg GAE/100 g [75], values much higher for red onion; and 103 mg GAE/100 g [76], 428 mg GAE/100 g [77], and finally a maximum of 571 mg GAE/100 g [78]. More elevated levels may be reached depending on some experimental conditions reported below [79,80]. This pattern is corroborated by comparative studies between varieties (in mg/kg GAE) that show higher TPs in red onions (up to 1083.04 mg/kg), followed by yellow (441–455 mg/kg) and white (142–160 mg/kg), which are consistent with those described by Gebremeskel et al. [81] and Kavalcová et al. [82], who pointed out genotype and growing conditions. The same trend is observed for total flavonoids, varying from 8 to 18 mg QE/100 g [83,84] until reaching values of 165.8 (mg QE/100 g) [78], 366 (mg QE/100 g) [82], and even 1376 (mg QE/100 g) [79], which is particularly significant in peels that are known to be one the richest sources of these compounds. Lastly, the condensed tannins, 4.99 mg CE/100 g [82] and 9.82 mg CE/100 g [76], present variability linked to the origin of the material, extraction conditions, and genetic and climatic factors. Therefore, the values obtained for onion peels indicate that we should not be preoccupied with biological variation but rather with the organ–variety–methodology combination when comparing with similar tissues and protocals whenever possible.

The HPLC/ESI-MS profile of red *A. cepa* peel extracts, by comparison, is characterized by a phytochemical fingerprint in which flavonols, predominantly organized by quercetin and its glycosylated derivatives, are accompanied by similar contributions from isorhamnetin. This supports the previous description of *A. cepa*, with quercetin and related phenolics (protocatechuic/vanillic acids and hydroxybenzoic derivatives) being metabolites identified by Lachman et al., 2003 [85], in addition to comparative profiles of onions (red, yellow, and white), which report quercetin as a major glycoside along with spiraeoside and rutin, as observed in Pudzianowska et al., 2012 [86]. The strong tendency of presenting glycosylated forms in our peels is also consistent with quercetin occurring in free and bound forms [87] and the numerous quercetin monoglycosides (including spiraeoside and positional glucosides) commonly identified in onions [88]. Isorhamnetin, a family of flavonols, has been previously reported to be characteristic of both red and yellow *A. cepa* varieties (albeit in glycosylated forms) [89].

At the organ level, the literature shows that *A. cepa* peels have the highest concentrations of quercetin and its derivatives, making them a valuable polyphenolic co-product [90]. Similarly, Burri et al. [91] detected, in the peel/skin of a variety of onions, a range of flavonols that were quercetin-based and also quercetin glucosides (mono/di/tri glucosides), kaempferol 3-*O*-glucoside, and isorhamnetin 3-*O*-glucoside, alongside phenolic acids like p-hydroxybenzoic and vanillic. Numerically, quercetin content in red *A. cepa* peels can be very high; the investigation by Senan et al. [92] reported that the Yemeni red onion peel contained about 52,289.14 µg/g of quercetin with chlorogenic acid (18,761.89 µg/g) and some gallic acid (1509.43 µg/g). The presence of phenolic acids together with the flavonolic pool strengthens the idea that a combined electron/hydrogen donating/chelating mechanism might be responsible for potent antioxidant activity, which may complement or act additively with flavonols [90]. Lastly, the finding of an anthocyanoside like cyanidin 3-*O*-glucoside is consistent with profiles of red onion peels, where anthocyanins may coexist with flavonols and influence antioxidant activity despite their contribution being dependent on stability and processing conditions [90]. In general, our findings corroborate with the common sense that red onion peels are a specially rich matrix in quercetin (and glycosides), isorhamnetin, and phenolic acids, while differences across studies are significantly influenced by plant genotype and growing conditions, agro-climatic conditions, and extraction/analysis conditions [90,92].

The different hierarchy of DPPH/FRAP and TAC could be also explained by the fact that these methods do not exactly measure the same dimension of antioxidation; whereas DPPH and FRAP assays mainly reflect a radical scavenging/electron transfer power, TAC corresponds to a total capacity (depending on reagent chemistry, kinetics, reagent concentration, and matrix). The highest activity of aqueous extracts or Soxhlet water would be due to a more effective extraction of polar substances with high reducing ability, and the superiority of hydroethanolic samples in TAC was attributed to an enrichment in components for which their antioxidant production is more beneficial in this test. This is solvent-sensitive, as alluded to by Kumar et al. [90]. In comparison, the DPPH IC_50_ values observed in our study are much lower than those published for red *A. cepa* by Nuutila et al., 2003 (67 mg/mL) [75] and Fredotović et al. [93], who reported a value of about 77.13 mg/mL. This difference is due to the matrix (peels are more concentrated than edible tissues), protocols, and the manner of expressing results. These results are in agreement with Senan et al. [92], stating that the high concentrations of quercetin and phenolic acids (chlorogenic, gallic) in Yemeni red *A. cepa* peels justify their strong antioxidant activities. These findings validate that antioxidant rankings depend on the assay and extraction and support a multi-assay approach (DPPH, FRAP, TAC) for the interpretation of this relationship between activity and expected chemical families: flavonols (quercetin and isorhamnetin) and phenolic acids [90].

The antimicrobial activity of the *A. cepa* peel decoction has a selective spectrum, especially against Gram-negative bacteria (*E. cloacae* and *P. aeruginosa*), which were inhibited at very low MICs. This is remarkable in light of the fact that Gram-negative bacteria are often difficult for antimicrobial agents to target because they have an outer membrane and efflux systems. The effectiveness indicates that the membranes and intracellular targets are disrupted, as polyphenols (quercetin and derivatives) can influence membrane function, energy metabolism, and nucleic acid biosynthesis [90,94]. Our findings are consistent with previous studies, suggesting that antibacterial activity is highly dependent on the extraction method and composition. Lee et al. [94] reported that crude extracts using subcritical water extraction (SWE) inhibited *S. aureus* (0.7–1.1 log CFU/mL) at slightly lower efficacy than pure quercetin. Kim et al. [95] likewise reported antimicrobial activity in peels extracted in SWE against *Bacillus cereus*, which was attributed to quercetin and oxidation products. Peel matrices yield strong antibacterial responses; however, as with the encapsulation of active agents, the process and target combination are critical [90].

Importantly, however, quercetin is not always the dominating element of antimicrobial strength. Crnivec et al. [96] showed that ethanolic/freeze-dried extracts of onion skin powder possessed lower MICs compared to pure quercetin, thus proving the synergistic potential of phenolics in conjunction with other active substances. Similarly, Fredotović et al. reported [97] that glycosylated flavonols exhibited lower antimicrobial activity than whole-peel extracts; this indicates the involvement of other phytocompounds. This variability emphasizes that some strains of bacteria could reduce the efficacy of crude extracts, particularly resistant ones such as MRSA, whereas others are still susceptible to polyphenol-induced membrane disruption or oxidative stress [98,99,100].

The low activity against terbinafine was expected for crude extracts, as polyphenols employed by the authors target wider mechanisms compared to targeted antifungal agents. However, the literature indicates that formulation strategies may enhance antifungal activity, including peel-derived green nanoparticles [101,102,103]. While less efficient than antibiotics, our observations of strong inhibition against Gram-negative bacteria warrant further studies on the mechanisms involved, fractionation (isolating specific active compounds), and synergisms with efflux inhibitors and membrane permeabilizers, as discussed in recent reviews on biomedical applications of onion peels [104,105,106].

The dose-dependent prolongation of PT and aPPT suggests a genuine anticoagulant effect, potentially consistent with the polyphenol-mediated modulation of hemostasis (effects on coagulation enzymes, redox balance, ion interactions, and platelet-related pathways), as previously reported for flavonoid-rich matrices [107,108]. Given the complexity of crude extracts, mechanistic refinement would benefit from pathway-focused assays and/or fractionation to identify the most contributive constituents relative to standards.

The inhibition results of α-amylase and α-glucosidase obtained with the decoction of *A. cepa* peels, for which their potency (EC_50_) exceeds that of acarbose, suggest a strong functional affinity of extract constituents toward these digestive enzymes, which is consistent with the richness of the peels in flavonols (quercetin and glycosides: spiraeoside, and isoquercitrin) and phenolic acids, and they are capable of interacting with proteins via hydrogen bond networks and hydrophobic interactions at the active site and/or peripheral sites, reducing carbohydrate hydrolysis and glucose release [109,110,111]. The bibliographic comparison clearly places these performances in the high range reported for onion peel/waste matrices, with several studies showing α-glucosidase inhibitions in the order of 1.27 and 0.15 mg/mL depending on the onion genotype and extraction solvent [112], emphasizing that the activity does not depend exclusively on quercetin but also on other bioactive fractions and synergy effects, as onion peel extracts can sometimes match or exceed standards (quercetin or acarbose) depending on experimental conditions, as demonstrated by Gois Ruivo da Silva et al., 2020 [113] and Nile et al. [113]. The in vitro/in vivo concordance reinforces the following mechanistic argument: The improvement of the OGTT (attenuation of the glycemic peak and decrease in AUC) is compatible with a slowing down of the hydrolysis/intestinal absorption of carbohydrates, and rat data also report a decrease in glycemic exposure after administration of onion peel extracts which is associated with inhibition of intestinal α-glucosidases [112], while other studies suggest additional mechanisms (improvement of insulin sensitivity, modulation of inflammation and oxidative stress) that can complement the enzymatic effect [114]. Finally, the absence of acute toxicity at 2 g/kg constitutes an initial signal of safety margin for a crude extract, while justifying, in accordance with development practices, subchronic evaluations including biochemistry, hematology, and histology to consolidate the safety profile [90].

In silico investigations (docking, MD, and MM-PBSA) converge with the experimental bioactivities and suggest a multi-target potential of major flavonoids in the decoction, particularly quercetin 4′-O-glucoside (4QG). Docking is best interpreted as a prioritization tool, whereas MD results (RMSD/RMSF, Rg SASA over 100 ns) support overall complex stability with target-dependent local adjustments, consistent with binding-site plasticity. MM-PBSA ranking indicated a marked thermodynamic preference for the Q4G-2CDU complex, driven mainly by favorable van der Waals contributions despite polar solvation penalties, a common pattern in polyphenol–protein binding [115,116]. Collectively, these computational results provide a rational mechanistic link between the flavonol-rich composition of onion peels and the observed multi-mechanism effects, while remaining consistent with the literature describing onion peels as a flavonol/glycoside-rich resources with broad bioactivity potential [117].

## 5. Conclusions

This study supports the hypothesis that Moroccan *A. cepa* peels can be valorized as a safe, phenolic/flavonoid-rich agro-industrial by-product with extraction-dependent bioactivity, including antioxidant capacity, selective antimicrobial effects, and antidiabetic potential, with in silico modeling providing mechanistic plausibility for major constituents. These conclusions are limited to Moroccan materials, and the extraction of onions peels in general should be carried out cautiously, as peel color and post-harvest/processing factors may affect composition and potency. Future work should prioritize batch-to-batch standardization, bioavailability/pharmacokinetics, chronic safety, and clinically relevant efficacy endpoints.

## Figures and Tables

**Table 1:** *A. cepa*’s origin, portion used, location, and harvesting season.

Scientific Name	Part Collected	Region	Province	Municipality	Latitude (x)	Longitude (y)	Altitude (m)	Time of Collection
*A. cepa* L.	Peels	Fez-Meknes	Boulemane	Guigou	33°21′55″ N	4°49′34″ W	1748 m	April 2025

**Table 2:** Extraction coding.

Methods of Extraction	Solvents	Codification
Soxhlet	Ethanol/Water (70/30; *v*/*v*)	E2
	Water	E1
Decoction	Water	E0

**Table 3:** Collection of evaluated fungal and bacterial strains accompanied by references.

Strains		Abbreviations	References
Gram-positive cocci	*Staphyloccocus epidermidis*	*S. epidermidis*	5994
	*Staphyloccocus aureus BLACT*	*S. aureus* 2510	4IH2510
	*Staphyloccocus aureus STAIML/MRS/mecA/HLMUP/BLACT*	*S. aureus* 2220	2DT2220
	*Streptococcus acidominimus*	*S. acidominimus*	7DT2108
	*Streptococcus group D*	*S. group D*	3EU9286
	*Streptococcus agalactiae*	*S. agalactiae*	7DT1887
	*Streptococcus porcinus*	*S. porcinus*	2EU9285
	*Enterococcus faecalis*	*E. faecalis*	2CQ9355
	*Enterococcuss faecium*	*E. faecium*	13EU7181
Gram-negative bacilli	*Acinetobacter baumannii*	*A. baumannii* 2404	7DT2404
	*Acinetobacter baumannii*	*A. baumannii* 2410	7DT2410
	*Escherichia coli*	*E. coli*	3DT1938
	*Escherichia coli* ESBL	*E. coli* ESBL 2057	2DT2057
	*Escherichia coli* ESBL	*E. coli* ESBL 5765	2DT5765
	*Enterobacter aerogenes*	*E. aerogenes*	07CQ164
	*Enterobacter cloacae*	*E. cloacae* 317	02EV317
	*Enterobacter cloacae*	*E. cloacae* 2280	2DT2280
	*Citrobacter koseri*	*C. koseri*	3DT2151
	*Klebsiella pneumoniae* ssp. *pneumoniae*	*K. pneumoniae* 1823	3DT1823
	*Klebsiella pneumoniae* ssp. *pneumoniae*	*K. pneumoniae* 1015	3DT1015
	*Proteus mirabilis*	*P. mirabilis*	2DS5461
	*Pseudomonas aerugenosa*	*P. aerugenosa* 2138	2DT2138
	*Pseudomonas aerugenosa*	*P. aerugenosa* 1124	2DT1124
	*Pseudomonas fluorescence*	*P. fluorescence*	5442
	*Pseudomonas putida*	*P. putida*	2DT2140
	*Serratia marcescens*	*S. marcescens*	375BR6
	*Salmonella* sp.	*Salmonella* sp.	2CG5132
	*Shigella* sp.	*Shigella* sp.	7DS1513
	*Yersinia enterocolitica*	*Y. enterocolitica*	ATCC27729
Yeasts	*Candida albicans*	*C. albicans*	Ca
	*Candida kefyr*	*C. kefyr*	Cky
	*Candida krusei*	*C. krusei*	Ckr
	*Candida parapsilosis*	*C. parapsilosis*	Cpa
	*Candida tropicalis*	*C. tropicalis*	Ct
	*Candida dubliniensis*	*C. dubliniensis*	Cd
	*Saccharomyces cerevisiae*	*S. cerevisiae*	Sacc
Fungi	*Aspergillus niger*	*A. niger*	AspN

**Table 4:** Molecular docking parameters and protein targets.

Activities	Targets	PDB ID	Grid Box Center Coordinates	Grid Box Size
Antibacterial activity	Sortase	4TQX	center_x = 22.976 center_y = 28.166 center_z = −23.161	size_x = 25 size_y = 28 size_z = 30
	DNA gyrase	1KZN	center_x = 18.325 center_y = 30.783 center_z = 36.762	size_x = 20 size_y = 38 size_z = 38
	Dihydropteroate synthase	2VEG	center_x = 31.404 center_y = 48.530 center_z = 0.204	size_x = 24 size_y = 24 size_z = 20
	Enoyl-[acyl-carrier-protein] reductase [NADPH] FabI	4FS3	center_x = 110.708 center_y = 67.251 center_z = 31.071	size_x = 35 size_y = 36 size_z = 38
	D-Alanine Ligase	2ZDQ	center_x = 47.378 center_y = 12.782 center_z = 5.730	size_x = 23 size_y = 26 size_z = 32
	Dihydrofolate Reductase complexed with novel 7-aryl-2,4-diaminoquinazolines	3SRW	center_x = −4.701 center_y = −31.536 center_z = 6.341	size_x = 26 size_y = 28 size_z = 23
	Penicillin-binding protein 1a PBP1a	3UDI	center_x = 34.198 center_y = −1.249 center_z = 12.715	size_x = 24 size_y = 24 size_z = 28
	Beta-lactamase	4K0X	center_x = 8.812 center_y = 6.668 center_z = 24.384	size_x = 25 size_y = 28 size_z = 26
	Carbapenem-hydrolyzing beta-lactamase KPC	4ZBE	center_x = 3.005 center_y = 2.027 center_z = 12.341	size_x = 26 size_y = 42 size_z = 34
	Transcriptional activator protein lasR	3IX3	center_x = 10.469 center_y = 4.844 center_z = 21.344	size_x = 27 size_y = 25 size_z = 30
	Beta-lactamase	4KZ5	center_x = 48.494 center_y = −1.379 center_z = 22.865	size_x = 36 size_y = 35 size_z = 40
	Enoyl-[acyl-carrier-protein] reductase [NADH]	4ZJU	center_x = 22.162 center_y = 15.963 center_z = 10.616	size_x = 25 size_y = 27 size_z = 28
	Topoisomerase IV subunit B	1S16	center_x = 33.628 center_y = 53.299 center_z = 2.203	size_x = 40 size_y = 38 size_z = 40
	Beta-lactamase NDM-1	4HL2	center_x = −4.007 center_y = −5.025 center_z = 16.291	size_x = 26 size_y = 34 size_z = 38
Antifungal activity	CYP51 VARIANT1	5FSA	center_x = 205.007 center_y = 14.197 center_z = 58.034	size_x = 28 size_y = 24 size_z = 32
	Lanosterol 14-alpha demethylase	5V5Z	center_x = −44.007 center_y = −14.161 center_z = 22.011	size_x = 24 size_y = 30 size_z = 36
Antioxidant activity	Lipoxygenase-3	1N8Q	center_x = 26.014 center_y = 0.014 center_z = 16.108	size_x = 22 size_y = 28 size_z = 32
	Cytochrome P450 2C9	1OG5	center_x = −38.207 center_y = 61.001 center_z = 27.024	size_x = 22 size_y = 28 size_z = 32
	NADPH oxidase	2CDU	center_x = 18.26 center_y = −6.350 center_z = −1.530	size_x = 24 size_y = 22 size_z = 28
	Xanthine dehydrogenase/oxidase	3NRZ	center_x = 58.097 center_y = 3.009 center_z = 35.108	size_x = 20 size_y = 28 size_z = 34
	Superoxide Dismutase	1HL5	center_x = 27.097 center_y = 111.039 center_z = 64.117	size_x = 22 size_y = 30 size_z = 32
	Glutathione peroxidase 1	2F8A	center_x = −8.001 center_y = 20.237 center_z = 19.842	size_x = 26 size_y = 28 size_z = 30
Antidiabetic activity	Pancreatic alpha-amylase	4W93	center_x = −9.004 center_y = 22.197 center_z = −17.361	size_x = 28 size_y = 22 size_z = 36
	Alpha-glucosidase	3W37	center_x = 16.169 center_y = −19.023 center_z = −31.110	size_x = 20 size_y = 22 size_z = 28
Anticoagulant activity	Thrombin Heavy Chain	4UFD	center_x = 13.087 center_y = −1.004 center_z = 18.0964	size_x = 24 size_y = 24 size_z = 30

**Table 5:** *A. cepa* peels’ moisture content, pH, and ash content.

Species	Moisture Content MC (%)	pH	Ash Content (%)
*A. cepa*	13.78 ± 0.69	3.06 ± 0.15	15.21 ± 0.76

**Table 6:** FAO/WHO maximum limits (2009) and heavy metal concentration (mg/kg).

Species	Lead (Pb)	Arsenic (As)	Chromium (Cr)	Iron (Fe)	Titanium (Ti)	Cadmium (Cd)	Antimony (Sb)
*A. cepa*	0.0968	0.1198	0.0655	0.6786	0.0729	0.0437	0.1223
Maximum limits (FAO/WHO)	3	1	2	20	−	0.3	1

**Table 7:** Phytochemical test results.

Compounds/Species		*A. cepa*
Part used		Peels
Sterols and triterpenes		++
Flavonoids		+++
Tannins	Catechic tannins	++
	Gallic tannins	+
Anthracene derivatives	Quinones	−
	O-Heterosides	−
	C-Heterosides	−
Saponosides		−
Oses and holosides		+++
Alkaloids	Dragendorff	++
	Mayer	++

Category: Strong presence: +++; average presence: ++; low presence: + and absent: −.

**Table 8:** Compounds identified by mass spectrometry in the peels of *A. cepa*.

RT (min)	Molecules	Classes	Exact Masses	[M–H]^−^ (*m*/*z*)	Fragment Ions (*m*/*z*)	Area %
3.46	Gallic acid	Phenolic acid	170	169	169-125	0.65
3.96	Ferulic acid	Phenolic acid	194	193	178-149-134	1.29
4.21	Gallocatechin	Flavonoid	306	305	305-287-261-179-125	0.59
4.82	syringaldehyde	Phenolic compounds	182	181	181-166-151-123	2.84
6.60	Chlorogenic Acid	Phenolic acid	354	353	353-191-179-173-135	0.87
7.77	Cyanidin 3-*O*-glucoside	Flavonoid	484	483	303-241-285	8.41
10.67	Isorhamnetin	Flavonoid	316	315	315-300-271-255-151	30.26
15.62	Gentisic acid	Phenolic acid	154	153	153-109-108	0.79
15.93	Alliospiroside A	Saponin	708	707	707-563-545-401	0.58
16.24	(−) epicatechin	Flavonoid	290	289	245-205-179-225	2.45
18.78	Gallic acid hexoside	Phenolic acid	332	331	331-271-211-169	2.85
20.35	trans-resveratrol	Polyphenol	228	227	227-185-159-143	2.20
21.72	Quercetin 4′-*O*-glucoside	Flavonoid	464	463	463-301-271-179	12.42
22.37	B-type procyanidin	Flavonoid	578	577	425-407-289-451-125	3.89
23.08	Patuletin	Flavonoid	332	331	331-316-301-271-151	4.01
24.21	Glabrol	Flavonoid	392	391	391-323-269-255	1.98
25.01	Quercetin	Flavonoid	302	301	301-179-151-273-271	14.03
25.35	Hyperoside	Flavonoid	464	463	463-301-271-179	4.61
25.59	Quercetin-3-*O*-xyloside	Flavonoid	434	433	433-301-271	2.28
25.97	Ellagic acid	Phenolic acid	302	301	283-257-229-185	3.00

**Table 9:** The percentages of phenolic compound classes identified in the *A. cepa* decoction.

Classes	Percentages (%)
Flavonoids	84.93
Phenolic acids	9.45
Phenolic compounds	2.84
Polyphenols	2.2
Saponins	0.58
Total	100

**Table 10:** The MIC, MBC, and MFC (µg/mL) values of the *A. cepa* decoction extract, as well as the MIC values of antibiotics and antifungal drugs.

Microorganism		*A. cepa*		Antibiotics *					Antifungals ^#^
		MIC	MBC or MFC	Gentamycin	Amoxicillin–Clavulanate	Vancomycin	Trimethoprim–Sulfamethoxazole	Penicillin G	Terbinafine
GPC	*S. epidermidis*	150	300	2		>8	>4/76		
	*S. aureus BLACT*	300	600	<0.5		2	<10		
	*S. aureus STAIML/MRS/mecA/HLMUP/BLACT*	2500	2500	>8		>8	>4/76		
	*S. acidominimus*	600	600	≤250		<0.5		0.03	
	*S. group D*	2500	2500	>1000		<0.5		0.13	
	*S. agalactiae (B)*	150	300	≤250		>4		0.06	
	*S. porcinus*	1200	1200	≤250		<0.5		0.06	
	*E. faecalis*	5000	5000	≤500		1	≤0.5/9.5		
	*E. faecium*	5000	5000	≤500		>4	>4/76		
GNB	*A. baumannii*	2500	5000	≤1	≤2/2		≤1/19		
	*A. baumannii* 2410	2500	5000	>8	>32/2		>8/152		
	*E. coli*	600	1200	2	8/2		≤1/19		
	*E. coli* ESBL	600	1200	2	>8/2		>4/76		
	*E. coli* ESBL 5765	*1200*	*1200*	>16	>32/2		>320		
	*E. aerogenes*	1200	2500	≤1	8/2		≤1/19		
	*E. cloacae*	75	150	>4	>8/2		>4/76		
	*E. cloacae* 2280	*75*	*150*	>16	>32/2		>8/152		
	*C. koseri*	2500	5000	≤1	>8/2		<20		
	*K. pneumoniae*	300	600	≤1	≤2/2		≤1/19		
	*K. pneumoniae* 1015	600	600	8	>32		40		
	*P. mirabilis*	300	600	2	≤2/2		>1/19		
	*P. aeruginosa*	75	150	2	>8/2		4/76		
	*P. aeruginosa* 1124	150	150	>4	>8/2		>4/76		
	*P. fluorescence*	600	1200	4	>8/2		4/76		
	*P. putida*	2500	2500	>4	>8/2		>4/76		
	*S. marcescences*	300	600	4	>8/2		>4/76		
	*Sallemonella* sp.	2500	2500	>4	8/2		>4/76		
	*Shigella* sp.	2500	2500	>4	8/2		>4/76		
	*Y. enterolitica*	1200	2500	≤1	8/2		2/38		
Yeasts	*C. albicans*	5000	>5000						12.500
	*C. kefyr*	5000	>5000						25.000
	*C. krusei*	>5000	>5000						50.000
	*C. parapsilosis*	5000	>5000						6.250
	*C. tropicalis*	>5000	>5000						12.500
	*C. dubliniensis*	>5000	>5000						3.125
	*S. cerevisiae*	1200	2500						3.125
Molds	*A. niger*	5000	>5000						3.125

*: The minimum inhibitory concentration (MIC) of the antibiotics (µg/mL) was ascertained using the BD Phoenix™ identification and antibiogram instrument (Becton, Dickinson and Company, Franklin Lakes, NJ, USA); ^#^: the MIC (µg/mL) of terbinafine was evaluated on a microplate.

**Table 11:** Protein targets and molecular docking specifications.

Activities	Targets\Ligands	Isorhamnetin	Quercetin	Quercetin 4′-*O*-glucoside	Cyanidin 3-*O*-glucoside
Antibacterial activity	4TQX	−6.7	−6.8	−6.6	−6.5
	1KZN	−7.6	−8.1	−8.2	−7.6
	2VEG	−7.5	−7.1	−8.1	−9
	4FS3	−9	−9.4	−9.8	−9
	2ZDQ	−8.6	−8.5	−9.4	−8.6
	3SRW	−8.5	−9	−9.3	−8.9
	3UDI	−8	−8.1	−9	−9
	4K0X	−7.7	−7.6	−8.1	−7.8
	4ZBE	−7	−7.4	−8.2	−7.6
	3IX3	−9.9	−9.8	−9.1	−7.1
	4KZ5	−7.6	−7.5	−8.3	−6.5
	4ZJU	−8	−8.4	−9.1	−9.4
	1S16	−8	−8	−8.2	−7.9
	4HL2	−7.5	−7.6	−7.8	−6.7
Antifungal activity	5FSA	−8.1	−8.9	−9.7	−8.6
	5V5Z	−8.3	−8.7	−8.8	−9
Antioxidant activity	1N8Q	−9.2	−9.3	−7.4	−9.4
	1OG5	−8.2	−8.2	−9.1	−8.2
	2CDU	−8.3	−8.4	−10.5	−9
	3NRZ	−8.1	−8.3	−8.7	−8.9
	1HL5	−7.9	−7.9	−8.4	−8.8
	2F8A	−6	−6.2	−6.3	−6.6
Antidiabetic activity	4W93	−7.9	−7.8	−8.2	−7.7
	3W37	−6.5	−6.7	−6.6	−6.7
Anticoagulant activity	4UFD	−8.5	−8.7	−8.7	−8.7

**Table 12:** 2D and 3D interaction profiles of Quercetin 4′-*O*-glucoside with target proteins.

Activities	Targets\Ligands	2D	3D
Antibacterial activity	4FS3	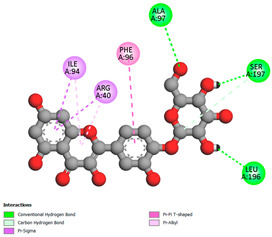	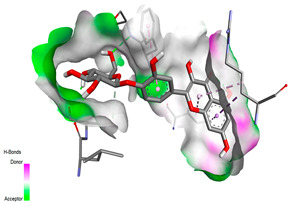
Antifungal activity	5FSA	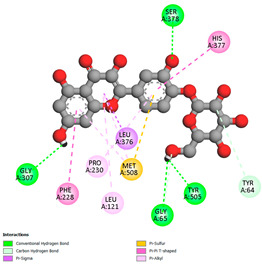	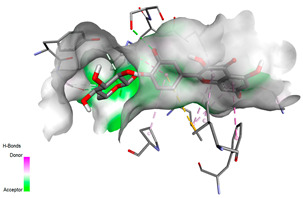
Antioxidant activity	2CDU	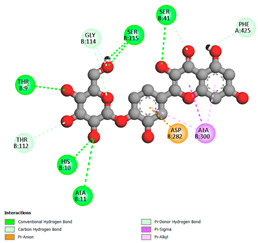	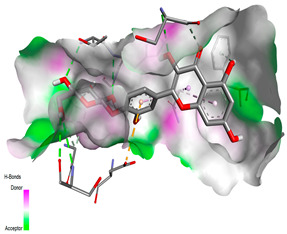
Antidiabetic activity	4W93	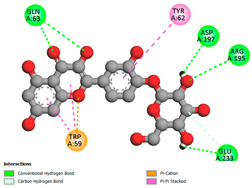	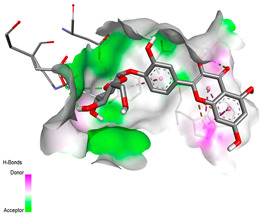
Anticoagulant activity	4UFD	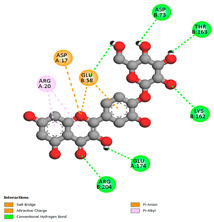	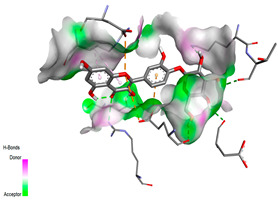

**Table 13:** MM-PBSA calculations were used to obtain the binding free energy in kcal·mol^−1^ for the systems under study.

Complex	Q4G-2CDU	Q4G-5FSA	Q4G-4FS3	Q4G-4UFD	Q4G-4W93
ΔE__VDW_	−81.66 ± 0.47	−47.67 ± 0.19	−45.92 ± 0.31	−37.83 ± 0.28	−40.94 ± 0.25
ΔE__EEL_	−48.40 ± 1.66	−25.86 ± 0.66	−33.27 ± 0.90	−65.62 ± 1.10	−64.54 ± 0.59
ΔE__PB_	67.63 ± 1.51	62.35 ± 0.60	59.41 ± 0.68	93.02 ± 1.04	99.33 ± 0.54
ΔE__NPOLAR_	−7.35 ± 0.02	−5.10 ± 0.01	−4.89 ± 0.02	−4.53 ± 0.02	−4.40 ± 0.01
ΔG__GAS_	−130.06 ± 1.68	−73.53 ± 0.62	−79.19 ± 0.91	−103.45 ± 1.07	−105.48 ± 0.53
ΔG__SOLV_	60.28 ± 1.52	57.25 ± 0.60	54.52 ± 0.67	88.49 ± 1.03	94.93 ± 0.53
ΔG__TOTAL_	−69.77 ± 0.78	−16.27 ± 0.42	−24.67 ± 0.41	−14.96 ± 0.45	−10.55 ± 0.44

## Data Availability

The original contributions presented in this study are included in the article. Further inquiries can be directed to the corresponding authors.
